# Benzophenone assisted UV-activated synthesis of unique Pd-nanodendrite embedded reduced graphene oxide nanocomposite: a catalyst for C–C coupling reaction and fuel cell†

**DOI:** 10.1039/c9ra02431j

**Published:** 2019-07-09

**Authors:** Teresa Aditya, Jayasmita Jana, Sonali Panda, Anjali Pal, Tarasankar Pal

**Affiliations:** Department of Chemistry, Indian Institute of Technology Kharagpur-721302 India tpal@chem.iitkgp.ac.in; Department of Civil Engineering, Indian Institute of Technology Kharagpur-721302 India

## Abstract

In this work we report the use of benzophenone (BP) for the synthesis of a palladium (Pd) embedded on reduced graphene oxide (rGO) nanocomposite (Pd/rGO) using a simple aqueous solution and UV irradiation. The simple and facile evolution of thermodynamically unstable branched Pd(0) nanodendrites was achieved by BP photoactivation, circumventing the growth of more stable nanomorphologies. The synthesis of Pd(0)-embedded rGO nanosheets (PRGO-nd) was made possible by the simultaneous reduction of both the GO scaffold and PdCl_2_ by introducing BP into the photoactivation reaction. The nanocomposites obtained in the absence of BP were common triangular and twinned Pd(0) structures which were also implanted on the rGO scaffold (PRGO-nt). The disparity in morphologies presumably occurs due to the difference in the kinetics of the reduction of Pd^2+^ to Pd^0^ in the presence and absence of the BP photoinitiator. It was observed that the PRGO-nd was composed of dense arrays of multiple Pd branches around nucleation site which exhibited (111) facet, whereas PRGO-nt showed a mixture of (100) and (111) facets. On comparing the catalytic efficiencies of the as-synthesized nanocatalysts, we observed a superiority in efficiency of the thermodynamically unstable PRGO-nd nanocomposite. This is due to the evolved active facets of the dendritic Pd(0) morphology with its higher surface area, as testified by Brunauer–Emmett–Teller (BET) analysis. Since both PRGO-nd and PRGO-nt contain particles of similar size, the dents and grooves in the structure are the cause of the increase in the effective surface area in the case of nanodendrites. The unique dendritic morphology of the PRGO-nd nanostructures makes them a promising material for superior catalysis, due to their high surface area, and the high density of surface atoms at their edges, corners, and stepped regions. We investigated the efficiency of the as-prepared PRGO-nd catalyst in the Suzuki–Miyaura coupling reaction and showed its proficiency in a 2 h reaction at 60 °C using 2 mol% catalyst containing 0.06 mol% active Pd. Moreover, the electrochemical efficiency for the catalytic hydrogen evolution reaction (HER) was demonstrated, in which PRGO-nd provided a decreased overpotential of 68 mV for a current density of 10 mA cm^−2^, a small Tafel slope of 57 mV dec^−1^ and commendable stability during chronoamperometric testing for 5 h.

## Abstract 2

Benzophenone photoinitiator aided synthesis of Pd-nanodendrite embedded rGO nanocatalyst possessing superior potential in C–C coupling reaction and fuel cell application.

## Graphical abstract

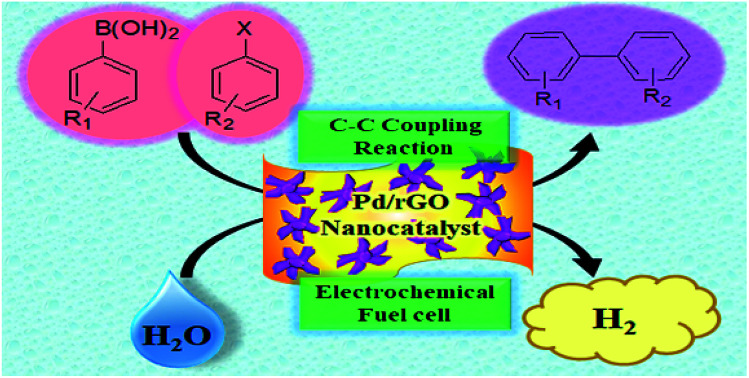

## Introduction

Nanomaterials have prompted momentous research due to their unique properties and applications in various fields including energy conversion and storage, chemical engineering, biological applications, environmental remediation and catalysis.^1–4^ The synthesis of advanced nanomaterials has been made possible by technologies which enable not only the preparation of tunable compositions, shapes, sizes and structures but also their detailed characterizations. Nanoparticles (NPs) containing a small percentage of noble metals that act as catalytically active sites when distributed on a support for maximum utilization of size, shape and crystal facets, are a current focus due their low cost syntheses.^5–7^

Palladium (Pd) NPs are a unique class of heterogeneous catalyst and are used in a wide range of research fields as sensors, devices, energy storage materials^5,7^ and catalysts of organic synthesis reactions, including the Suzuki–Miyaura, Sonogashira, Stille and Heck carbon–carbon coupling and carbon–oxygen bond formation reactions.^9,10^ Pd NPs as heterogeneous catalysts for the synthesis of chemical compounds have advantages over homogeneous catalysts with respect to stability, selectivity, cost-effectiveness, reusability and the presence of multiple active sites.^11–13^ Moreover, Pd NPs, if not stabilized, tend to agglomerate due to their high surface area-to-volume ratios, which may deactivate the catalyst. To overcome the cons of homogenous Pd catalysts, promising strategies have been undertaken to immobilize and stabilize the Pd NPs in various heterogeneous supports to maximize the active surface area of the catalysts, and so enhance catalytic activity and durability. Among available choices, graphene oxide (GO) is a common flexible support for such heterogeneous catalyst synthesis.

Graphene, a two-dimensional (2D) atomic layer of sp^2^-hybridized carbon atoms, has been proposed as a novel class of catalyst due to its high specific surface area, outstanding electrical conductivity, superior mechanical flexibility, good chemical/thermal stability and capability for functionalization.^14,15^ It has been widely shown that graphene-based catalysts increase the catalyst surface area for electron transport and provide better mass transport of reactants. However, graphene is hydrophobic in nature due to its lack of functional groups which limits its usefulness for the universal immobilization of NPs. In contrast, graphene derivatives like GO and reduced GO (rGO) provide a stable catalyst structure and exhibit hydrophilicity due to their surface functional groups which enable the facile attachment of metal NPs.^16,17^ The syntheses of metal/rGO catalysts by one-pot co-reduction have been widely studied leading to new and modified synthetic procedures. Moreover, novel strategies involving alternative reagent and energy inputs are expected to lead to desirable changes in the morphology and composition of the heterogenous catalysts, resulting in novel chemical and catalytic properties. In this context the UV irradiation-assisted synthesis of Pd NPs has not been widely studied and there have been few reports till date. For the first time, we have meticulously manipulated the morphology of Pd(0) on rGO sheets by the addition of the initiator benzophenone (BP) under UV irradiation. To the best of our knowledge, using a photoinitiator-assisted synthetic approach to embed nanodendritic Pd NPs in rGO has not previously been reported.

Carbon–carbon coupling reactions facilitated by metal catalysts are crucial reactions that have been extensively explored. Among all coupling reactions, the Suzuki–Miyaura Coupling Reaction, which was first reported in the 1970s, is the most versatile and commonly used method for C–C bond formation.^18–21^ Suzuki–Miyaura Coupling is a convenient technique as it can be performed in a biphasic, aqueous medium or without solvent. A wide range of water-soluble bases, catalyst systems, and reagents can be employed. The reaction process involves the coupling of organoboron compounds (organoborane, organoboronic acid, organoboronate ester or potassium trifluoroborate) with aryl, alkenyl or alkynyl halides. The reaction commonly gives good yields in the temperature range of 25–100 °C. This reaction has the edge over other cross-coupling methods such as the Heck reaction or Stille reaction, due to its milder reaction conditions and its use of boronic acids and their derivatives that are environmentally benign when compared to other organometallic reagents. Furthermore, when compared to other organometallic reagents, particularly in the case of large-scale synthesis, the management and removal of boron-containing by-products can be conveniently achieved. The catalytic cycle revolves around oxidative addition, transmetalation and reductive elimination steps. Usually, the reaction is carried out using homogeneous catalysts with different Pd complexes which poses a problem during recovery,^9,21^ and hence heterogeneous catalysts have attracted attention.^11,12,22^ Nonetheless, there are pressing issues related to such systems, such as the high activation barriers of substrates which are related to the rate-limiting steps, the requirement for elevated temperatures (≥100 °C) or reflux conditions, and time consuming processes, which may lead to undesired side reactions and the disruption of catalyst stability.^1,23^ However, the coupling reaction is scalable and is a commercial methodology for manufacturing intermediates for pharmaceuticals and fine chemicals. Moreover, large-scale synthesis has been successful for a number of important biological compounds including potential central nervous system agent.

The impending exhaustion of fossil fuels has led to a momentous leap in research on sustainable, abundant and eco-friendly energy systems. Hydrogen has emerged as the ideal energy carrier in term of its high specific storage density and abundance in nature.^2,24–26^ Hydrogen does not exist naturally on the Earth's surface but can be obtained from compounds such as hydrocarbons and water by three primary methods: steam–methane reforming, coal gasification and water electrolysis. While other methods have the limitation of significant carbon dioxide (CO_2_) emission causing pollution, hydrogen derived from photocatalytic or electrocatalytic water splitting is an alluring alternative renewable source for energy conversion and storage due to its compliance with the reduction of pollution and global warming.^24,27,28^ Water decomposition comprises two steps: the oxygen evolution reaction (OER) and the hydrogen evolution reaction (HER), but due to the sluggish kinetics of these processes, prospective commercialization is hampered. To solve this problem, novel catalysts which can improve the efficiency of the reactions are imperative. For this purpose platinum (Pt) based catalysts are the most profoundly studied, but the scarcity and cost of Pt and the low stability of the catalysts impede wide-scale application. The next in line, with respect to efficiency, is Pd with its higher abundance, lower cost and high efficiency. Catalysts of Pd metal and Pd-based compounds in the form of composites, alloys, hybrids, intermetallics, bimetallics, *etc.*, are well-known for fuel cell applications and have been studied extensively.^27–29^

This work reports a radically different approach for Pd/rGO nanomaterial synthesis, adopting a facile one-pot protocol and simple reaction setup, resulting in a unique, rarely reported nanodendritic structure. The as-synthesized nanomaterial is subsequently applied in the Suzuki–Miyaura coupling reaction and electrochemical HER. To the best of our knowledge, there are no prior reports of the synthesis of such Pd nanodendrites embedded on rGO, with novel kinetically controlled enhanced surface area, in an aqueous medium using benzophenone system under UV irradiation. Our method evades the use of polymer, surfactant and heat treatment, thus producing a potential heterogeneous catalyst for multifaceted applications.

## Experimental section

### Synthesis of PRGO-nt and PRGO-nd

In a typical synthesis, two vials were employed, and to each vial 1 × 10^−3^ M PdCl_2_ solution was added into an aqueous suspension of 10 mg GO. To one of the vials, 1 mL ethanol without BP was then added and to the other vial, 1.5 × 10^−2^ M BP dissolved in 1 mL ethanol was introduced. The total volume of the reaction mixture was maintained at 10 mL in each case and the vials were irradiated with UV light. The vials were removed from UV exposure after 6 h, and the solids were washed with water and ethanol and dried for further use. The product obtained from ethanol alone was labelled PRGO-nt and that yielded from BP was termed PRGO-nd. Another pair of reactions having the same reaction mixtures was subjected to treatment in our modified hydrothermal (MHT) reactor. The sample prepared without BP was termed PRGO-a and that with BP was termed PRGO-b. All the products were washed with water and ethanol, and reserved for the next course of experiments.

### Pd/rGO NPs as a catalyst for the Suzuki–Miyaura coupling reaction

In a typical reaction, 2 mol% of Pd/rGO NPs, 0.5 mmol of aryl halides, and 0.55 mmol of phenyl boronic acid were added into a round bottom flask. Then, 2 mmol of K_2_CO_3_ and 4 mL water–ethanol (v/v = 1 : 1) were added into the flask. The flask was sealed and the mixture was stirred at 60 °C for 2 h. The catalyst was then separated from the mixture using a short column (silica gel). The pure product was obtained by work-up with ethyl acetate using a separating funnel, and dried over excess anhydrous Na_2_SO_4_. Then the solvent was removed in a rotary evaporator and the crude product was purified by column chromatography on silica gel (hexane/ethyl acetate) to obtain the isolated product. The structures of the pure products were confirmed by ^1^H NMR and ^13^C NMR spectroscopy.

### Recyclability of the catalytic system

To test the recyclability of the catalyst for a set of reactions, a reaction mixture containing the catalyst was filtered and washed repeatedly with ethyl acetate until no trace of any reactants were found in the filtrate using thin layer chromatography (TLC) paper. The catalyst was then washed with water several times to check the complete removal of K_2_CO_3_ using litmus paper, and finally washed a few more times with ethanol. The catalyst was then dried under vacuum pressure and re-used for the next cycle.

### Electrocatalytic activity of the PRGO catalyst in HER for fuel cell

The PRGO-nd and PRGO-nt catalysts were exploited for electrochemical investigation to obtain an insight into the effect of morphology on catalysis. The electrocatalytic measurements were carried out with a three electrode cell in an electrochemical workstation, using a glassy carbon electrode (GCE), geometric area 0.07 cm^2^, coated with the sample catalyst as the working electrode, a platinum wire with a diameter of 1 mm as the counter electrode and a saturated calomel electrode (SCE) as the reference electrode. For the HER study, GCEs were drop-cast with 7 μL of the as-prepared PRGO-nd or PRGO-nt, or a commercial dispersion of Pt/C (1 mg mL^−1^ concentration) individually, and dried in air for 3 h. This resulted in a catalyst loading of 7 μg. On top of the loaded GCE, Nafion solution was applied as a binder. The supporting electrolyte used was 0.5 M H_2_SO_4_ solution in water. Linear sweep voltammetry (LSV) was conducted by sweeping the potential from 0.0 to −0.2 V (*vs.* RHE) at a scan rate of 5 mV s^−1^ in the electrolyte solution under 700 rpm magnetic stirring. Electrochemical impedance spectroscopy (EIS) was executed at a bias potential of −0.068 V (*vs.* RHE), in a frequency range of 0.1 Hz to 10^5^ Hz with an AC voltage of 5 mV amplitude. The potential selected for chronoamperometric investigation was −0.068 V (*vs.* RHE), which resulted in a current density of 10 mA cm^−2^ for 5 h. All the potentials reported in this study were normalized with respect to the reversible hydrogen electrode (RHE) using the equation *E*(RHE) = *E*(SCE) 0.242 + 0.059 pH (V).

## Results and discussion

**Fig. 1:**
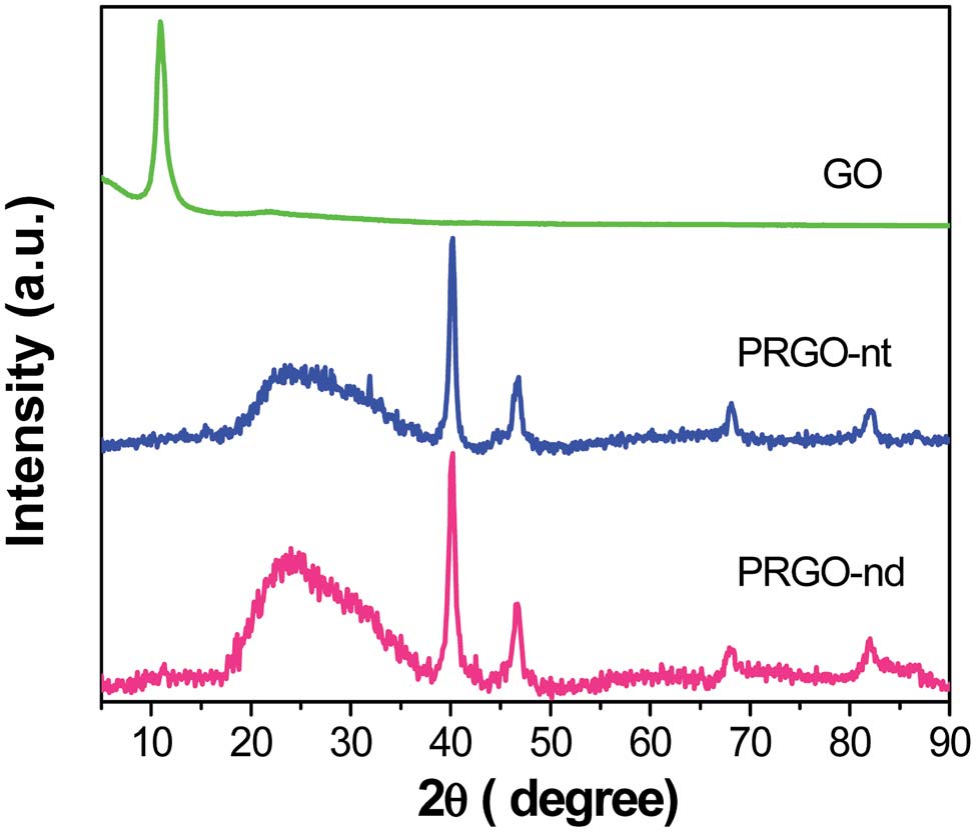
XRD analysis comparing GO, PRGO-nt and PRGO-nd.

The XRD patterns of PRGO-nt and PRGO-nd (Fig. 1) show peaks which can be indexed to Pd(0). The peaks at 2*θ* values of 40.3, 46.8, 68.1 and 82.0° can be indexed to the corresponding XRD patterns from the (111), (200), (220) and (311) lattice planes of a face-centred cubic (fcc) Pd crystalline structure (JCPDS no. 05-0681). We observed a peak of pure GO at 10.7° which vanished after reduction during the synthesis and a new peak at 24.7° was generated. For the samples prepared with and without BP, the peak positions were identical, proving that the compounds synthesized are alike in chemical composition.

**Fig. 2:**
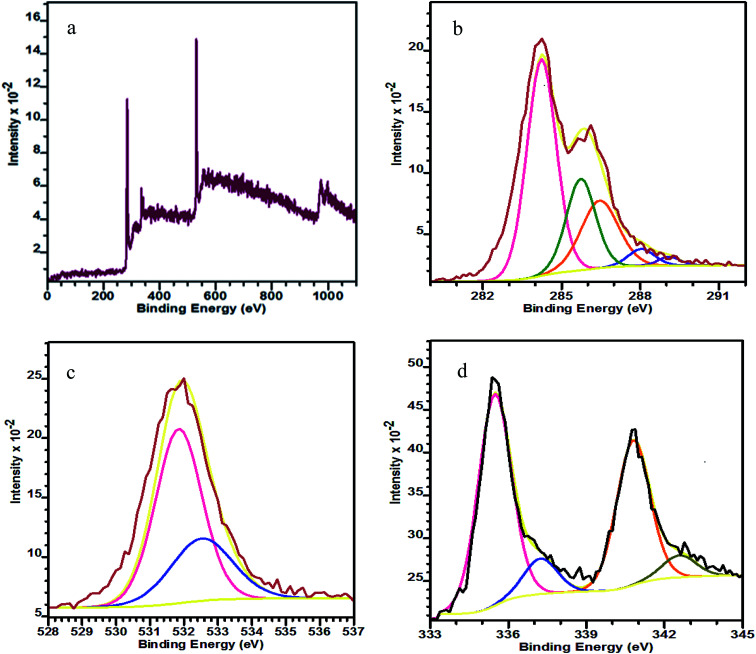
XPS spectra of PRGO-nd: (a) wide-scan, (b) C 1s, (c) O 1s, and (d) Pd 3d.

The wide-scan XPS spectrum in Fig. 2a shows the presence of C, O and Pd elements in the as-prepared PRGO-nd nanocomposite. Two pairs of asymmetric intense peaks (Fig. 2d) are evident in the Pd 3d spectrum which can be attributed to Pd(0). On deconvolution, these give weak peaks could be attributed to Pd(ii) species such as PdO and Pd(OH)_2_.^30^ The two deconvoluted peaks at 335.6 and 337.2 eV can be indexed to Pd 3d_5/2_, and are at lower binding energies than those of the Pd 3d_3/2_ peaks at 340.8 and 342.6 eV.^31,32^ The C 1s XPS spectrum (Fig. 2b) has a main peak at 284.2 eV which is characteristic of the sp^2^-hybridized C of graphitic material. The deconvolution of the C 1s spectrum gives peaks for C–C bonds at 285.7 eV, C–O at 286.4 eV, C=O at 288.0 eV and O–C=O at 289.1 eV.^33–35^ The O 1s peak (Fig. 2c) at 531.09 eV can be indexed to C–OH and that at 532.43 eV to rGO.^36^

**Fig. 3:**
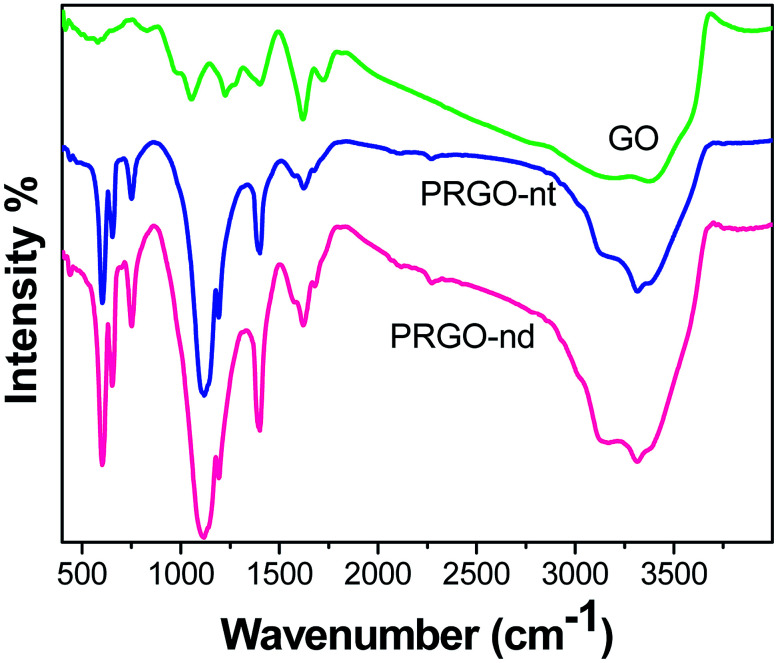
Comparison of FTIR spectra for GO, PRGO-nt and PRGO-nd.

The FTIR spectra (Fig. 3) obtained of the Pd/rGO products were investigated. In the spectrum of pure GO, the presence of C–O is detected at 1052 cm^−1^, and bending vibrations of C–OH appear at 1417 cm^−1^. The C=C skeletal vibrations are noted at 1626 cm^−1^. The peak for C=O vibrations occurs at 1736 cm^−1^ and its intensity decreases as the GO is reduced to rGO in both PRGO-nd and PRGO-nt.^37^ The peak of O–H stretching is observed at 3403 cm^−1^, and peaks at 800 and 1060 cm^−1^ are also caused by OH.^38^ In rGO, the intensities of all these oxygen-containing functional bands are reduced indicating that the reduction has taken place.^39^ C–C stretching vibrations are observed at 1580 cm^−1^ and those of C–O are at 1100 cm^−1^ in rGO.^40^

**Fig. 4:**
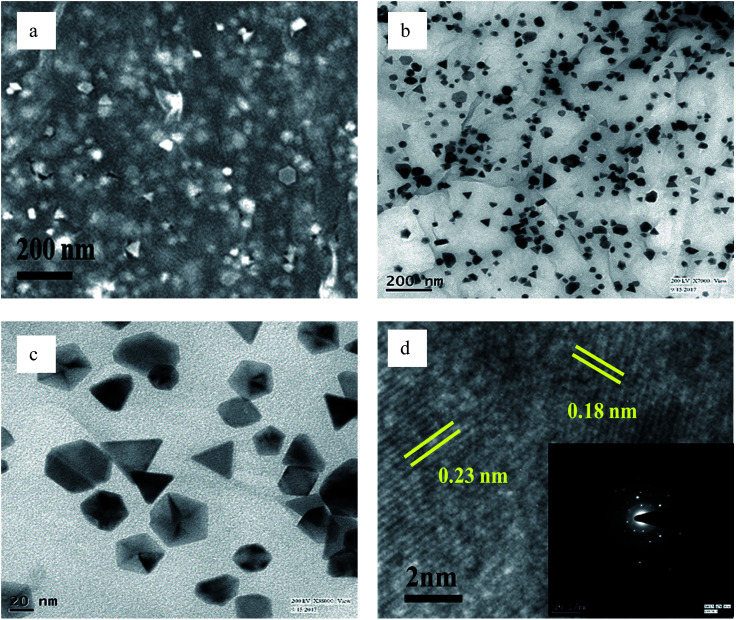
(a) FESEM, (b and c) TEM, and (d) HRTEM images of PRGO-nt (The inset of part d shows the SAED pattern).

**Fig. 5:**
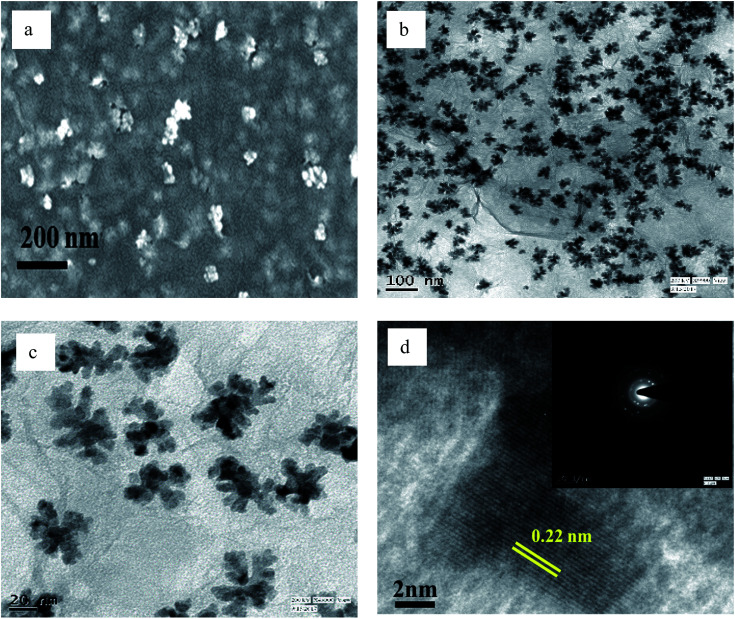
(a) FESEM, (b and c) TEM, and (d) HRTEM images of PRGO-nd (The inset of part d shows the SAED pattern).

The FESEM and TEM images of pure GO show large 2D sheets around 20-30 μm in length (Fig. S1†). The GO, which was synthesized by the classical Hummers' method,^41^ was brownish in colour. The compound was easily dispersed in aqueous medium. All the as-prepared compounds were dispersed with sonication before investigation thereby proving the robustness of their structures. The Pd(0) particles distributed on the rGO sheets are triangular when prepared in the presence of ethanol without BP (Fig. 4), while in the presence of ethanol with, the structures are branched (Fig. 5).

Usually, the thermodynamically favorable structures of fcc noble metals like Pd nanocrystals are cuboctahedra and multiple twinned particles. When PdCl_2_ is reduced at a high rate by ethanol to produce Pd(0) atoms, the final product is characterized by these thermodynamically favored shapes owing to their relatively low surface free energies.

However, when the reduction is substantially slower, the nucleation and growth is governed by kinetic control and the final NPs can be tailor-made to display a range of nanodendritic structures which deviate from the thermodynamically favored shapes.^7^ In the presence of BP, the Pd(0) NPs are nanodendritic structures. Thus, the presence of BP, ethanol and UV give conditions for the growth of such nanodendritic structures. Under identical condition in the absence of graphene, ill-defined structures of Pd(0) are obtained (Fig. S2†). We observed that the product in ethanol in the absence of BP contains mainly triangular plates of about 30–40 nm in length, along with cuboctahedra and twinned NPs of length 40–50 nm. The fringe spacings were measured to be 0.18 nm for the (100) facets and 0.23 for the (111) facets which are consistent with the selected area electron diffraction (SAED) pattern. In the case of the BP-assisted synthesis, the product exhibits branched structures of length 30–40 nm. Random branches are formed which are attached at the middle and look like dendrites. The fringe spacing is 0.22 nm for (111) facet, consistent with the SAED pattern. When the amount of BP was changed (3 × 10^−3^ M and 3 × 10^−2^ M), the exclusive formation of nanodendrites was disrupted and ill-defined Pd(0) particles which do not reveal any unique morphology are found distributed in the rGO sheets. The MHT subjected PRGO-a shows structures similar to those of PRGO-nt, whereas MHT subjected PRGO-b exhibited cubic and rectangular structures as the major morphology. We also investigated the hydrothermal products and observed mixed faceted structures having (100) and (111) facets (Fig. S3a†) in the absence of BP, whereas the presence of BP lead to (100) facet only (Fig. S3b†).

**Fig. 6:**
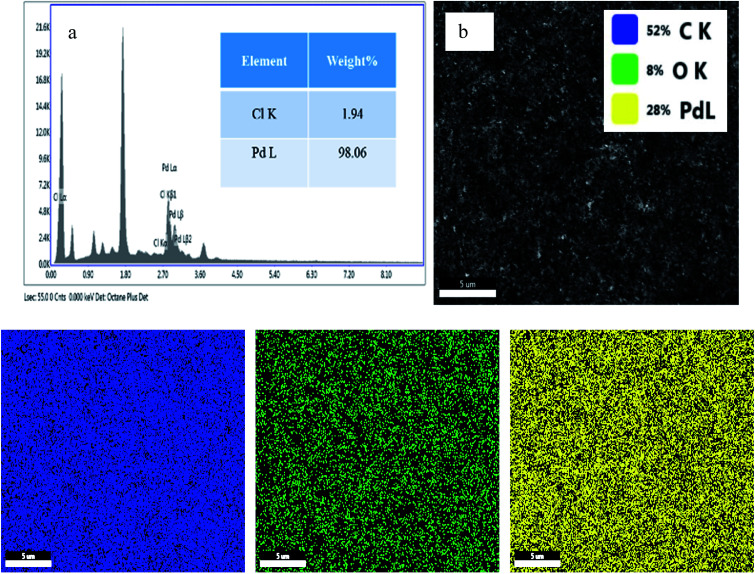
(a) EDAX and (b and c) elemental mapping of PRGO-nd (The inset of part b shows the respective).

Energy-dispersive X-ray analysis (EDAX) and elemental mapping of both PRGO-nt and PRGO-nd were conducted. In the case of PRGO-nt (Fig. S4†), abundant Pd was converted from PdCl_2_ and low levels of Cl were evident on the as-prepared 2D nanocomposite. However, in comparison, PRGO-nd (Fig. 6) displays an improvement in Pd concentration and negligible Cl. In the case of PRGO-nd, both EDAX and elemental mapping show a much higher distribution of Pd than that observed in PRGO-nt. The elemental mapping of both the Pd/rGO samples show an even distribution of Pd particles throughout the rGO.

**Fig. 7:**
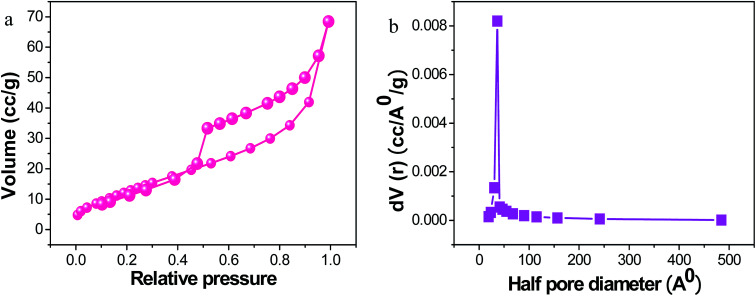
(a) BET analysis and (b) pore size distribution of PRGO-nd.

The Brunauer–Emmett–Teller (BET) analysis clearly shows the more extensive surface area of PRGO-nd at 51.82 m^2^ g^−1^ (Fig. 7) when compared to that of PRGO-nt at 34.43 m^2^ g^−1^ (Fig. S5†). The higher activity of PRGO-nd towards catalysis is an obvious implication. The pore size diameter obtained for PRGO-nd was 77.66 Å, and that for PRGO-nt was 73.25 Å. The higher surface area of the nanodendrites is probably due to grooves and dents in the structure resulting in a more exposed surface area than that of an even surface, even though both the PRGO-nt and PRGO-nd particles are of similar size. Therefore, the results are in accordance with the structures observed in the FESEM and TEM images. Hence, a greater surface area and the high density of surface atoms at their edges, corners and stepped regions, give a larger number of active sites for catalysis to the PRGO-nd, and corroborates it as a promising material for efficient catalytic activity in any given reaction.

### Syntheses of PRGO

In a typical synthesis, the PdCl_2_ was reduced on rGO in an aqueous medium using ethanol with BP and ethanol without BP in two separate cases. The product formed in the presence of BP had morphologically different Pd(0) species when compared to the product formed in the absence of BP. In both the cases, Pd(0) was uniformly deposited on the rGO sheets to give PRGO nanocomposites, as seen from the characterizations. The morphology of the products formed under UV irradiation is intriguing: Pd(0) formed by reduction in ethanol in absence of BP was triangular in shape and some particles showed twinned morphology (PRGO-nt); however, the Pd(0) deposited on rGO in the presence of BP photoinitiator takes the form of branched dendrites (PRGO-nd), which is a rare morphology for Pd. In the case of PRGO-nt, both a low and a high concentration of ethanol resulted in particles that are similar in size and morphology, but in the case of PRGO-nd, lowering the amount of BP disrupted the formation of the nanodendritic structure. Moreover, an excess of BP prevented the formation of nanodendritic particles (Fig. S6†) presumably due to heavy adsorption of BP during nucleation.

We next used TEM in order to investigate the fate of the products when the reaction mixtures were subjected to our modified hydrothermal (MHT) reactor (Fig. S3†). MHT treatment of the reaction aqueous mixture containing PdCl_2_, GO, BP and ethanol produced no dendritic structures. Rather it gave cubic and rectangular structures with (100) facets. However, in the absence of BP, the reaction mixture treated in the MHT reactor gave the same results as those obtained for UV irradiation.

**Scheme 1:**
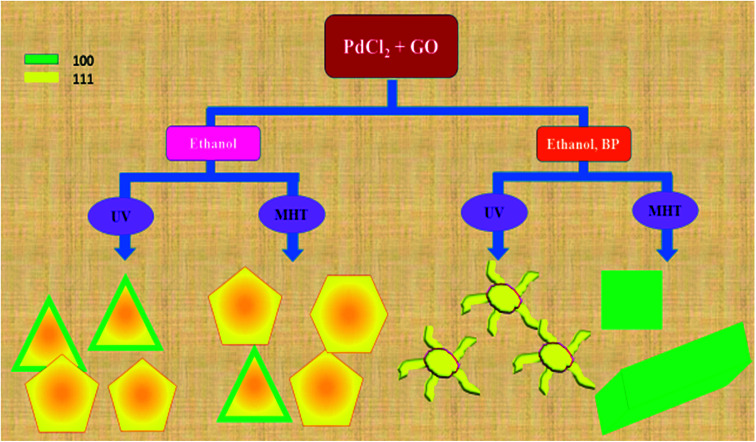
Schematic representation of products obtained using different reaction conditions during the syntheses of PRGO.

A schematic representation of the structures of Pd deposited on rGO sheets obtained under different reaction conditions is shown in Scheme 1. The schematic diagram shows the different types of reaction conditions applied to the reaction mixtures with and without BP, and the resulting structures and facets. Hence, it was established that the nanodendritic architecture was constructed exclusively under UV irradiation with the optimized amount of ethanol with BP. We tried a range of concentrations of BP and ethanol to confirm this conclusion. The nanocrystal size is a manifestation of the surface area and the percentage of surface atoms, while the shape or morphology is determined by the surface structure, the arrangement of atoms, and the sample composition. Apart from size, shape is a vital parameter which differentiates the properties of nanocomposites. Different morphologies and facets exhibit different physical and chemical properties, as in the case of Pd.^5–8,32^ The fcc crystal structure of Pd has various exposed facets depending on morphology. In the case of PRGO-nt we observed triangles, pentagons and hexagons with exposed (111) and (100) facets.^32,42–44^ Prior to our work, the role of photo-irradiated BP for controlling the morphology of Pd nanomaterials had not been recognized. Photo-irradiated BP causes the manifestation of the nanodendritic structure suppressing the thermodynamically stable shapes. The fcc metals have low-index surfaces with surface energies in the order (111) < (100) ≪ (110).^44^ After the initiation of nucleation of Pd(0) NPs in PRGO-nd, the growth favors (111) facet, which is distinct from the fringe spacing of the exposed dendrites. The facet having the highest surface energy tends to grow the fastest and hence, in time, vanishes from the final nanostructure as in the case of the PRGO-nd nanocomposite. Thus the (100) facet gets eradicated with gradual exposure of the (111) facet which has a slow growth rate and manifests itself in the final structure. However, the slow growth rate in the presence of BP and rGO hinders the formation of proportionate thermodynamically facile geometric shapes and results in dendritic structures.

**Scheme 2:**
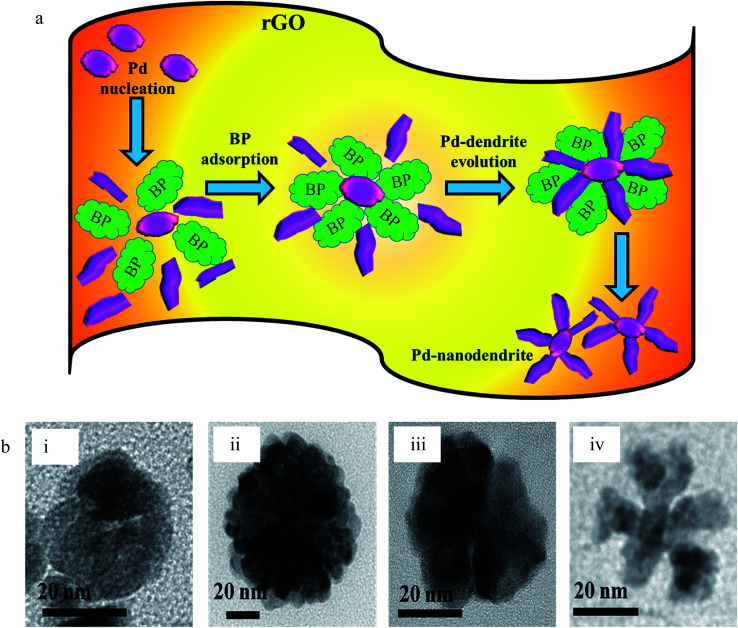
(a) Representative diagram of the mechanism of synthesis of PRGO-nd in the presence of BP. (b) TEM images of Pd NPs deposited on rGO using (i) acetone, (ii) hydroquinone, (iii) acetophenone, and (iv) BP.

We also presumed that the preferential adsorption of BP on Pd may have facilitated the final blossoming of the nanodendritic structures (Scheme 2a). To test our assumption, we carried out the reaction with acetone (Fig. S7a†), hydroquinone (Fig. S7b†) and acetophenone (Fig. S7c†) at the same molar concentration, substituting BP in the synthetic protocol of PRGO-nd. Comparative TEM images of the resulting products delivered different types of nanodendritic morphologies of Pd on rGO, as shown in Scheme 2b. As expected, nanodendritic structures are obtained with slight variation in the density of the array of nanodendrites formed. Fu *et al.* have previously reported that the amino acid arginine alters the reduction kinetics of a Pd precursor and is preferentially chemisorbed on Pd (111) facets which in turn directs the formation of Pd tetrapods.^45^ Moreover, Zhu *et al.* reported that highly branched Au NPs can be formed in the presence of GO nanosheets which act as the scaffold.^46^ We know that GO is a 2D honeycomb lattice monolayer of carbon atoms. It is noteworthy that its basal planes and edges are loaded with oxygen-containing functionalities like carboxyl, hydroxyl and epoxy groups. Hence, during reduction, the oxygen-containing moieties not only serve as embedding sites for the *in situ* nucleation of the metal but are also responsible for the subsequent diffusion-limited growth.^47^ Ill-defined metal nanocrystals are obtained when the reaction is repeated in the absence of GO nanosheets (Fig. S2†).

### Screening and scope of the Pd/rGO NP catalyzed Suzuki–Miyaura coupling reaction

The Suzuki–Miyaura coupling reaction is one of the most convenient methods for C–C coupling. The reaction does not require complex experimental setup and therefore is a facile and coveted protocol for carrying out important reactions. It has been widely exploited in chemicals, paints, pharmaceuticals *etc.*

**Scheme 3:**
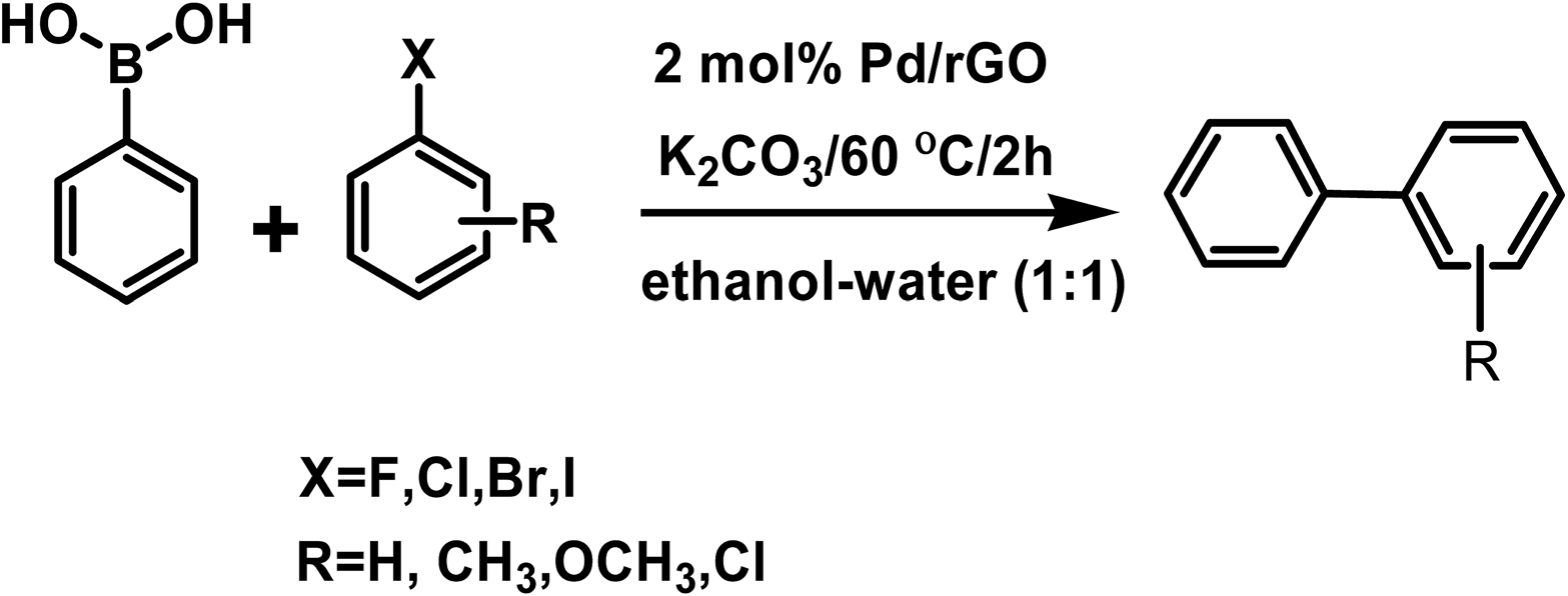
Scheme of the Suzuki–Miyaura coupling reaction.

**Scheme 4:**
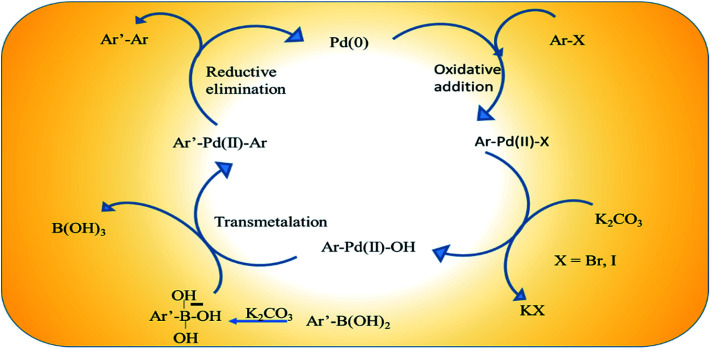
Scheme of the catalytic cycle in the Suzuki–Miyaura coupling reaction.

In our study we conducted the reaction using phenyl boronic acid and various unsubstituted/substituted aryl halides in the presence of a base in an ethanol–water (1 : 1) solvent system, at 60 °C for 2 h using only 2 mol% of the as-prepared catalyst (Scheme 3). The screening and control experiments are listed in Table 1. The reaction produced the desired products in yields that were good to almost quantitative, at 60 °C. In each of the reactions, only one product was obtained with no trace of by-products. No conversion was observed in the absence of base or the Pd/rGO nanocatalyst (entries 1, 2), which are essential for the activation of boronic acid and facilitate transmetalation. Aryl iodides and aryl bromides (entries 3, 5–9) were converted more readily than their chloride counterparts, as expected (entry 4). An electron withdrawing group in the aryl halide compound, particularly in the -*o* or -*p* positions, facilitated the reaction process as it assisted the rate-limiting oxidative addition step of the Pd catalytic cycle (Scheme 4), leading to high yields (entries 8, 9).^48^

**Table 1:** Suzuki–Miyaura coupling reaction conditions and yields using different aryl halide and phenyl boronic acids

Entry	X	R	Reaction condition variations	Temp (°C)	Yield (%)
1	Br	H	No K_2_CO_3_	60	—
2	Br	H	No catalyst	60	—
3	Br	H	2 mol% PRGO-nd, K_2_CO_3,_ EtOH/H_2_O (1 : 1), 2 h	60	81.0
4	Cl	H	2 mol% PRGO-nd, K_2_CO_3_, EtOH/H_2_O (1 : 1), 2 h	60	44.6
5	I	*o*-CH_3_	2 mol% PRGO-nd, K_2_CO_3_, EtOH/H_2_O (1 : 1), 2 h	60	98.0
6	I	*p*-CH_3_	2 mol% PRGO-nd, K_2_CO_3_, EtOH/H_2_O (1 : 1), 2 h	60	97.8
7	I	*p*-OCH_3_	2 mol% PRGO-nd, K_2_CO_3_, EtOH/H_2_O (1 : 1), 2 h	60	97.5
8	Br	*p*-Cl	2 mol% PRGO-nd, K_2_CO_3_, EtOH/H_2_O (1 : 1), 2 h	60	98.5
9	I	*p*-Cl	2 mol% PRGO-nd, K_2_CO_3_, EtOH/H_2_O (1 : 1), 2 h	60	98.7

The coupling of *o*-iodotoluene with phenyl boronic acid was chosen as a model reaction for studying the catalytic activity of Pd/rGO. The yield when using PRGO-nd was higher than those obtained with PRGO-nt, PRGO-a or PRGO-b (Table 2).

**Table 2:** Suzuki–Miyaura coupling reaction conditions and yields using *o*-iodotoluene and phenyl boronic acid

Entry	Catalyst (conditions of synthesis)	Reaction condition variations	Temp (°C)	Yield (%)
1	PRGO-a (MHT, without BP)	2 mol% catalyst, K_2_CO_3,_ 2 h, ethanol–water (1 : 1)	60	83.5
2	PRGO-b (MHT, in BP)	2 mol% catalyst, K_2_CO_3_, 2 h, ethanol–water (1 : 1)	60	88.4
3	PRGO-nt (UV, without BP)	2 mol% catalyst, K_2_CO_3_, 2 h, ethanol–water (1 : 1)	60	84.2
4	PRGO-nd (UV, in BP)	2 mol% catalyst, K_2_CO_3_, 2 h, ethanol–water (1 : 1)	60	98.0

We also used bromobenzene to compare the % yields of biphenyl product obtained with PRGO-nd (81%) and PRGO-nt (74%). This proved the potency of PRGO-nd with respect to the PRGO-nt nanocatalyst. The cause of the better activity of PRGO-nd is probably its higher surface area due to the grooves and channels in its structure. All the isolated products (biphenyl, 2-phenyltoluene, 4-phenyltoluene, 4-phenylanisole, 4-phenylchlorobenzene) were confirmed with ^1^H and ^13^C NMR (Fig. S9†).

### Insight into the Suzuki–Miyaura coupling reaction mechanism

The catalytic species is generated *in situ* directly from Pd(0) embedded on rGO.^49^Scheme 4 illustrates a proposed plausible reaction mechanism by considering the coupling of an aryl halide and aryl boronic acid with K_2_CO_3_. The adsorbed aryl halide molecules on the Pd/rGO NPs surface formed surface organo-palladium species *via* oxidative addition (Scheme 4). In the presence of base, the surface organo-palladium species become electrophilic intermediates, and this leads to transmetalation with the generation of B(OH)_3_, KHCO_3_ and KBr. Afterwards, the reaction is completed and the desired biaryl product is obtained by means of reductive elimination, which accompanies the restoration of the catalyst.

The high catalytic performance of the Pd/rGO catalyst can be attributed to its hybrid nanostructure and the synergistic effects of the individual components. Firstly, the high specific surface area of rGO is not only beneficial for the dispersion and stability of Pd NPs, but also propitious to the rapid diffusion of reactants. There is a π–π stacking interaction between rGO and aromatic aryl halide molecules, which helps the reactants to gain access to the Pd NPs more easily. The non-coplanarity of the two benzene rings in aromatic biaryl compounds also accelerates the reaction by forcing the product molecules away from the surface of rGO. Secondly, the steric configuration of the biphenyl and its solubility in the EtOH/H_2_O system further facilitate the reaction process. Moreover, the precipitation of biphenyl during the reaction process can drive the equilibrium to the product side. On account of polarity, the adsorption process takes place more easily between the hydrophilic rGO sheet and polarized substrates, especially the aryl boronic acid molecules, while the less polarized biaryl products readily desorb which further facilitates the product generation (entry 4).^50^ After repeated use in the reaction, the PRGO catalyst loses its activity due to the loss of Pd particles from the rGO scaffold, as observed in the TEM images recorded after 4 cycles (Fig. S8a†). This loss of Pd is also manifested in the corresponding decrease in intensity of the XPS spectrum of Pd (Fig. S8b†), which indicates the decrease of Pd content in the PRGO-nd catalyst after catalysis. From Table 3 we observe that this simple one-pot synthetic protocol to enhance surface area *via* the production of Pd-nanodendrites is scalable for gram scale use, and surpasses favorably to the other complicated syntheses of Pd-based catalysts which involve several steps, complex organic compounds and intricate set-ups. An added advantage of our catalyst is the small amount of active Pd(0) which is in play in the catalytic cycle of the coupling reaction. The Pd content was proven to be 3.28% in the PRGO-nd nanomaterial using the data from ICP-MS. The use of a minimal quantity of noble metal to make the reaction feasible has a strong bearing on the economic aspect of reactions which involve such expensive noble metals. Hence out of the 2 mol% total catalyst, only 0.06 mol% Pd actively participated in the reaction, making the catalyst superior in efficiency to other catalysts and economically attractive for application.

**Table 3:** Catalytic performances of different Pd-based catalytic systems for the Suzuki–Miyaura coupling reaction of *p*-iodoanisole and phenyl boronic acid^a^

Entry	Catalyst system used	Reaction conditions	Yield (%)
Ref. 51	Pd/MCoS-1 (0.2 mol% Pd)	K_2_CO_3_, H_2_O, 80 °C, 6 h	92
Ref. 52	Pd@g–C_3_N_4_–Fe-GO (2.5 mol%)	K_2_CO_3_, H_2_O/EtOH, 120 °C, 2.3 h	86
Ref. 53	PANI-Pd (0.14 mol% Pd)	K_2_CO_3_, H_2_O/dioxane, 95 °C, 4 h	89
Ref. 54	HMMS-salpr-Pd (1 mol%)	K_2_CO_3_, H_2_O/EtOH, 70 °C, 1 h	94
Ref. 55	Glucose-derived *in situ* Pd (1 mol%)	K_2_CO_3,_ H_2_O/^i^PrOH, 80 °C, 20 h	94
Ref. 56	Fe_3_O_4_@SiO_2_-TEGIL-Pd (0.025 mol%)	K_2_CO_3_, H_2_O, 60 °C, 7.5 h	97
Ref. 57	Fe_3_O_4_/IL/Pd (0.2 mol% Pd)	K_2_CO_3_, H_2_O/EtOH, r.t., 0.25 h	96
Ref. 58	Pd-Mont (0.07 mol% Pd)	K_2_CO_3_, H_2_O, 60 °C, 1 h	94
Ref. 59	GO/Fe_3_O_4_/Pd (0.5 mol% Pd)	K_2_CO_3_, H_2_O/EtOH, 80 °C, 0.25 h	95
Ref. 60	Pd/rGO (0.2 mol% Pd)	K_2_CO_3_, H_2_O/EtOH, 80 °C, 3 h	87
This work	PRGO-nd (0.06 mol% Pd in 2 mol% nanocomposite catalyst)	K_2_CO_3_, H_2_O/EtOH, 60 °C, 2 h	97.5

a Abbreviations used: MCoS-1 = porous Co(ii)-salicylate metal–organic framework, g-C_3_N_4_ = graphitic carbon nitride, PANI = polyaniline, HMMS = hollow magnetic (Fe_3_O_4_) mesoporous silica spheres, salpr = *N*,*N*′-bis(3-salicylidenaminopropyl)amine, TEGIL = triethylene glycol-imidazolium ionic liquid, IL = ionic liquid, Mont = montmorillonite clay.

### Fuel cell application

**Fig. 8:**
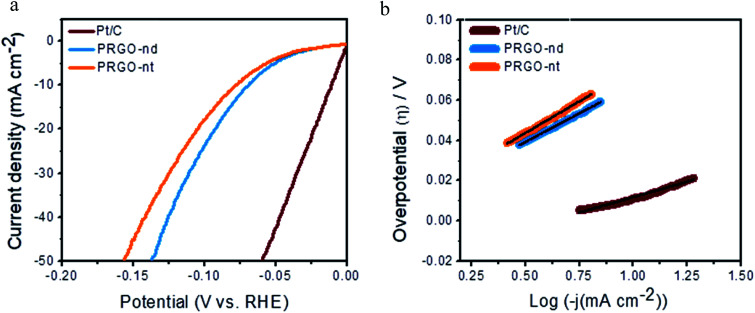
(a) HER polarization curves of the various as-prepared catalysts and commercial Pt/C. (b) Corresponding Tafel plots of the as-prepared catalysts compared to that of commercial Pt/C.

Sustainable production of hydrogen is a vital prerequisite for fuel cells and the hydrogen economy. The electrochemical HER is extensively studied and involves the production of hydrogen from proton reduction, using robust catalysts to decrease the overpotential and energy consumption of the reaction. Herein, we have investigated the catalytic HER to get insightful information on the morphological role of the PRGO-nd and PRGO-nt nanocatalysts in efficient hydrogen production from water splitting. The reaction was carried out in acidic electrolyte and was monitored using commercial Pt/C (Pt NPs at 20% mass loading) as the reference catalyst, which exhibited the highest activity with a negligible onset potential, a minimal overpotential of ∼11 mV to gain 10 mA cm^−2^ current density, and a Tafel slope of ∼30 mV dec^−1^. Linear sweep voltammetry (LSV) curves were obtained for both the PRGO-nd and PRGO-nt nanocatalysts using 0.5 M H_2_SO_4_ aqueous solution at a scan rate of 5.0 mV s^−1^ (Fig. 8a). The unloaded GCE showed a negligible current density at −0.5 V (*vs.* RHE). The onset potential was negligible for both the as-synthesized PRGO-nd and PRGO-nt catalysts and their catalytic efficiencies were also fairly comparable with that of the commercial Pt/C reference material. The overpotential observed to deliver 10 mA cm^−2^ current using PRGO-nd (68 mV) was better than that for PRGO-nt (77 mV). This could be attributed to the larger surface area available in the case of the nanodendritic Pd catalyst in comparison to the solid Pd triangles on the rGO scaffold. Linear portions of the Tafel plots were fitted to the Tafel equation, *η*_H_2_/2H^+^_ = *b* log *j* + *a*, where *η* is the overpotential, *b* is the Tafel slope, *j* gives the current density, and a is the empirical coefficient.^25,29^ The Tafel slopes were calculated to be around ∼57 mV dec^−1^ for PRGO-nd and ∼63 mV dec^−1^ for PRGO-nt which exhibits the intrinsic catalytic activity of the catalysts towards HER (Fig. 8b). The probable catalytic mechanism employed for accomplishing successful evolution of hydrogen is the Volmer–Heyrovsky mechanism which leads to the formation of surface-bound hydrogen molecules on the working electrode in the rate-determining step.^25,27^

**Fig. 9:**
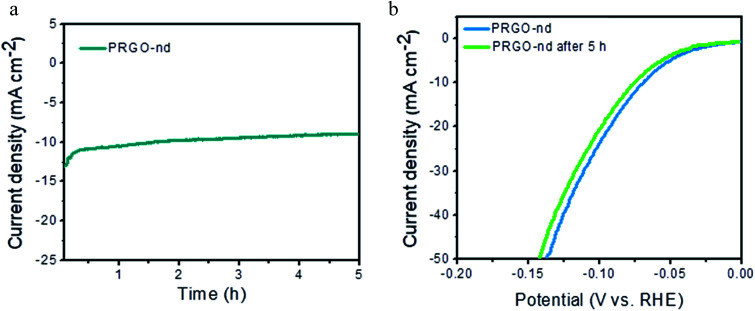
(a) Chronoamperometric test of PRGO-nd at −0.068 V (*vs.* RHE) for 5 h. (b) LSV curve of PRGO-nd before and after 5 h of testing.

The electrochemical capacitance surface area (ESCA) can be calculated from the electrochemical double layer capacitance (*C*_dl_) *i.e.* slope of the graph of capacitive currents at +0.2 V (*vs.* RHE) plotted against scan rate (Fig. S10†); the specific electrochemical double layer capacitance of the smooth GCE surface (*C*_s_) is 18 μF cm^−2^ in 0.5 M H_2_SO_4_ electrolyte in our case. From the equation, 
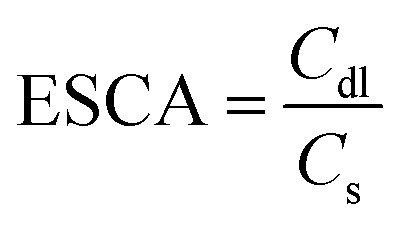
, the ESCA for PRGO-nd was determined as 47 cm^2^ and that for PRGO-nt was 44 cm^2^ which gives a clear sign of the enhanced catalytic activity of PRGO-nd for electrochemical HER. From the Nyquist plots produced from electrochemical impedance spectroscopy (EIS) (Fig. S11a†), obtained in 0.5 M H_2_SO_4_ solution by applying an AC voltage of 5 mV in a frequency range of 0.1 Hz to 10^5^ Hz recorded at a bias potential of −0.068 V (*vs.* RHE), we obtained the values for series resistance (*R*_s_) and charge transfer resistance (*R*_ct_) in the electrode/electrolyte interface and also the equivalent circuit diagram (Fig. S11b†). The *R*_s_ values were calculated to be 8.07 Ω, 8.52 Ω and 8.90 Ω, whereas the *R*_ct_ values were found to be 8.8 Ω, 12.5 Ω and 16.0 Ω for Pt/C, PRGO-nd and PRGO-nt, respectively. The lower *R*_ct_ value of PRGO-nd compared to that of PRGO-nt due to the smaller semi-circle diameter shows that there is a decrease in the charge transfer resistance at the electrode surface and implies that the PRGO-nd system is kinetically more favorable because, the Pd NPs embedded on the rGO sheets act as free electroactive adsorption sites enhancing the electron transfer in the system for efficient HER.^25–29^ The durability of the PRGO-nd catalyst was further tested using chronoamperometry (Fig. 9a) at a constant potential of −0.068 V (*vs.* RHE) and it showed potential stability with a small (∼10%) decay of activity after 5 h (Fig. 9b).

Hence from the above observations it is clear that PRGO-nd and PRGO-nt were both efficient as HER catalysts but the former showed a slightly better performance owing to the higher surface area of the PRGO-nd dendritic structure in comparison to the triangular morphology of PRGO-nt.

## Conclusion

To sum up, we have reported for the first time the use of BP photoinitiator as an agent which governs the slow growth and cause the evolution of exclusive (111) faceted Pd(0) nanodendrites in the Pd/rGO nanocomposite. The introduction of the photoinitiator causes a change to the thermodynamically stable triangle or twinned morphologies, as observed in PRGO-nt, which are most frequently reported. The variation in reaction kinetics give rise to a dendritic structure as observed in PRGO-nd, which is a rare morphology for Pd(0) NPs. The operational factors that produce the intriguing PRGO-nd morphology not only affect the stability of a particular facet but also the rate of growth of the Pd NPs after nucleation. We have elucidated the causes of the structure formation and have compared the catalytic efficiency of the PRGO-nd morphology with that of the more common thermodynamically favored structures. The multiple-branched architecture of PRGO-nd exposes larger active surfaces for the Suzuki–Miyaura coupling reaction which is accomplished at 60 °C in 2 h with 2 mol% catalyst containing 0.06 mol% active Pd. Furthermore, the high electrochemical efficiency of the PRGO-nd catalyst in HER was evident from the decreased overpotential of 68 mV for a current density of 10 mA cm^−2^, a small Tafel slope of 57 mV dec^−1^ and the commendable stability of the catalyst in chronoamperometric test. Therefore, our work describes a scalable synthetic protocol for a catalyst with inordinate potential, and opens doorway to stimulate further research on photoinitiator-assisted synthesis of metal nanocomposites.

## Conflicts of interest

There are no conflicts to declare.

## Supplementary Material

RA-009-C9RA02431J-s001
