# Supplementary material for: Benzophenone assisted UV-activated synthesis of unique Pd-nanodendrite embedded reduced graphene oxide nanocomposite: a catalyst for C–C coupling reaction and fuel cell
Source: RSC Adv. 2019 Jul 9;9(37):21329–43. doi: 10.1039/c9ra02431j (PMC9066186; doi:10.1039/c9ra02431j)
Supplement: RA-009-C9RA02431J-s001 [file RA-009-C9RA02431J-s001.pdf]

Electronic Supplementary Material (ESI) for RSC Advances.
This journal is © The Royal Society of Chemistry 2019

Benzophenone Assisted UV-activated Synthesis of Unique Pd-nanodendrite Embedded Reduced Graphene Oxide Nanocomposite: A Catalyst for C-C Coupling Reaction and Fuel cell

Teresa Aditya[a], Jayasmita Jana[a], Sonali Panda[a], Anjali Pal[b] and Tarasankar Pal[*,a]

[a]Department of Chemistry and [b]Department of Civil Engineering, Indian Institute of Technology, Kharagpur-721302, India

E-mail: tpal@chem.iitkgp.ac.in

Analytical instruments. Powder X-Ray diffraction (XRD) was investigated with a Philips PW-1710 X-ray diffractometer (40kV, 20 mA) using Cu Kα radiation (λ = 1.5418 Å) in the range of 5°-90° at a scanning rate of 0.5° min^{-1}. For analysing the XRD data, JCPDS software was referred. Surface morphology was examined using field emission scanning electron microscopy (FESEM) with a supra, Carl Zeiss Pvt. Ltd. Elemental detection of the nanomaterials was done with an energy dispersive X-ray microanalyser (OXFORD ISI 3000 EDAX) attached to the scanning electron microscopy. For acquisition of further information on structural aspect, transmission electron microscopy (TEM) analysis was done with the help of Hitachi H-9000 NAR 2 transmission electron microscope, using accelerating voltage at 200 kV. Surface chemical analysis was analysed by X-Ray photoelectron spectroscopy, XPS (SPECS PHOIBOS 100 MCD energy analyser), in an ultra-high vacuum environment (1.9 × 10−9 mbar) using Al Kα anode (1486.6 eV). Pass energy of 40 eV for survey scan and 30 eV for high resolution scan have been used during acquisition of the XPS spectra. Fourier transform infrared (FTIR) studies were performed with a Thermo-Nicolet continuum FTIR microscope. Raman spectra were recorded for the solid samples by using a fiber-coupled

micro-Raman spectrometer (Horiba Jobin Yvon Technology) equipped with 488 nm (2.55 eV) of 5 mW air cooled Ar^+ laser as the excitation light source, a spectrometer (model TRIAX550, JY), optical microscope (Model BX 41, Olympus, Japan), and a Peltier-cooled CCD detector (Horiba scientific, France). The BET analysis providing the N_2 adsorption−desorption isotherm was recorded at 77 K using Autosorb-iQ (Quantachrome Instruments). The ^{1}HNMR spectra were measured with Bruker-400 (400 MHz) or Bruker-500 (500 MHz) and ^{13}C NMR spectra were measured with Bruker-500 (125 MHz) using $CDCl_3$. Coupling constants in ^{1}H NMR are in Hz. The electrochemical measurements were studied using an electrochemical analyzer (CHInstruments Inc., CHI 660E).

Chemicals. All the chemicals used were of AR grade and used without purification. Double distilled water was used for all the experiments. Palladium Chloride ($PdCl_2$), Benzophenone (BP) were purchased from Sigma-Aldrich. All the organic solvents (hexane, ethyl acetate, dichloromethane) and acids were purchased from Sigma-Aldrich. $KMnO_4$, graphite powder, form E-Merck. Ethanol from Sisco Research Laboratory, India. Glass apparatus were cleaned with aqua regia, water and and double distilled water, and dried prior to use. Solvents were dried and distilled following usual protocols prior to use. All yields refer to isolated yields after column purification. Column chromatography was carried out using Silica gel (60-120 mesh) purchased from Rankem, India. TLC was performed on aluminium-backed plates coated with Silica gel 60 with F254 indicator (Merck).

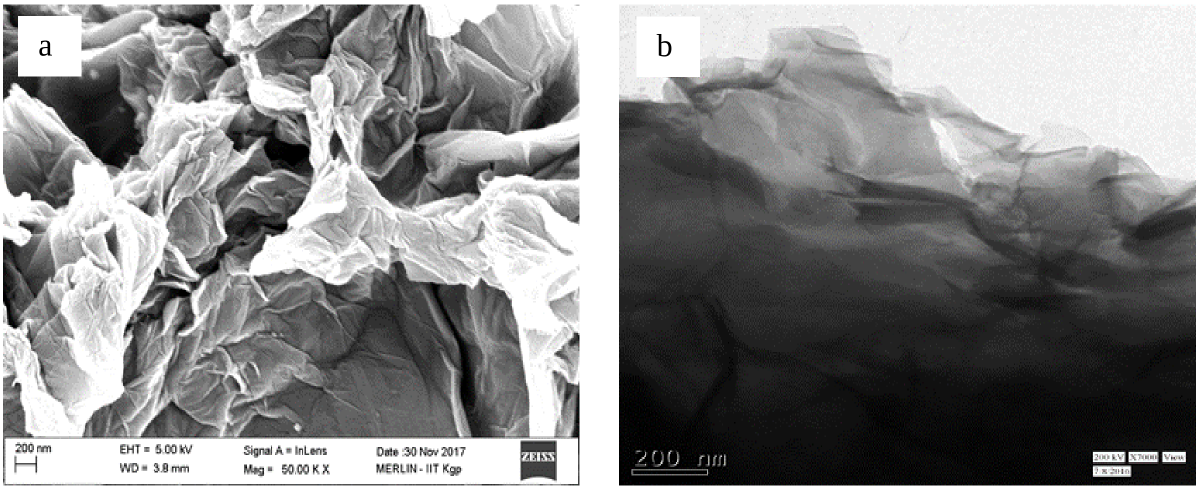

Figure S1. (a) FESEM and (b) TEM image of pure GO.

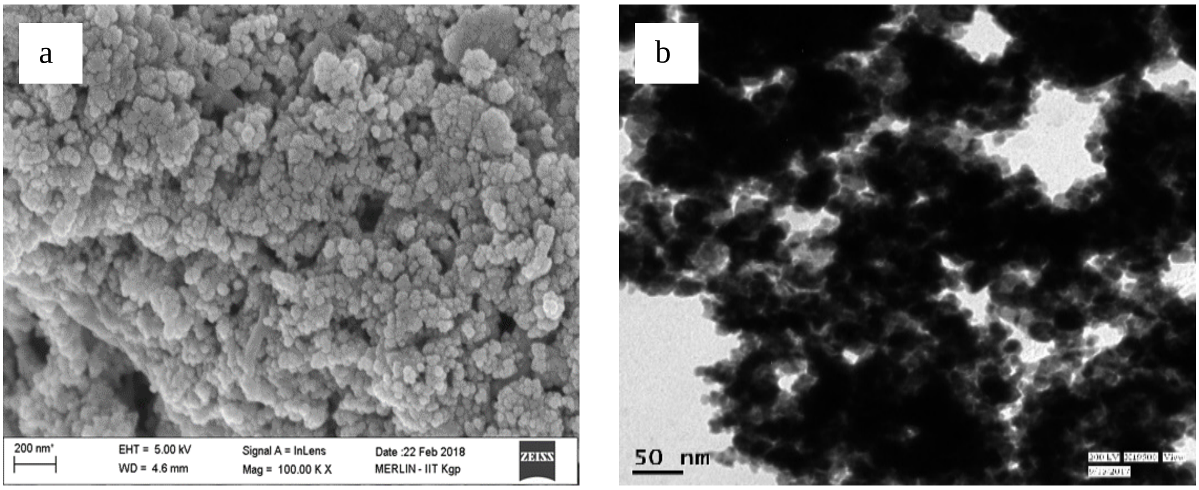

Figure S2. (a) FESEM and (b) TEM image of Pd(0) synthesized in ethanol with BP, in absence of GO, under UV-irradiation.

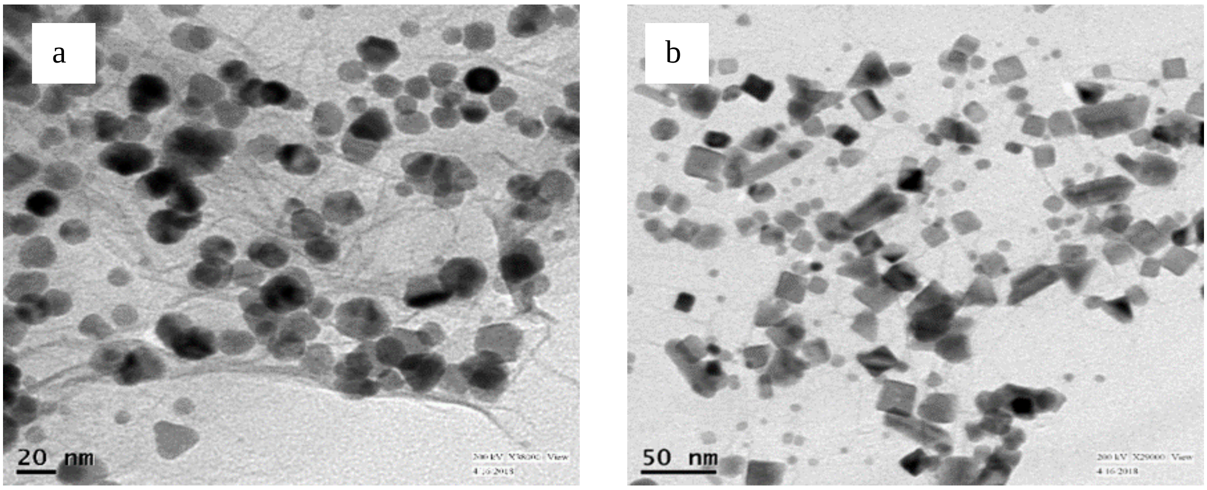

Figure S3. TEM images of Pd/rGO using $PdCl_2$ and GO under MHT using (a) ethanol without BP and (b) ethanol with BP.

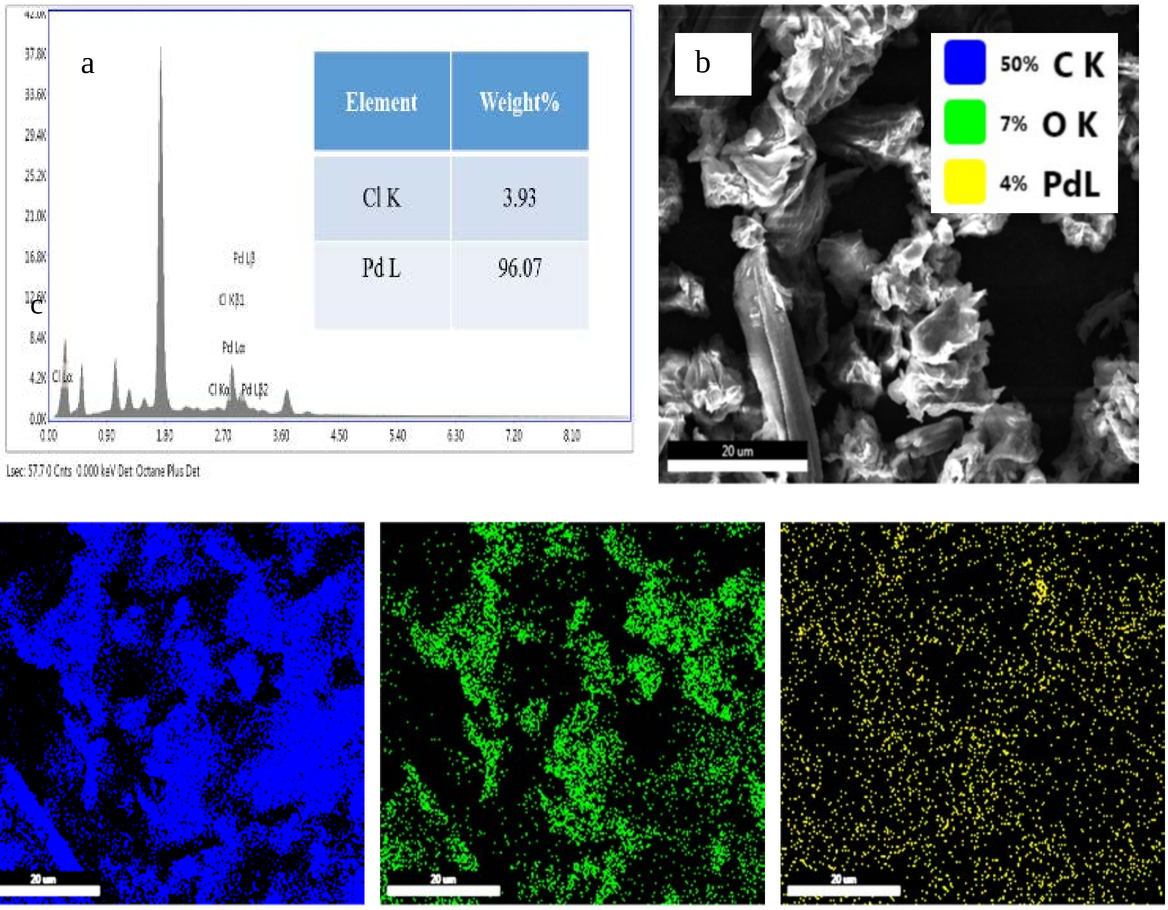

Figure S4. (a) EDX analysis and (b,c) elemental mapping of and PRGO-nt. (Inset: respective percentage of the elements of the corresponding colors denoted in part c).

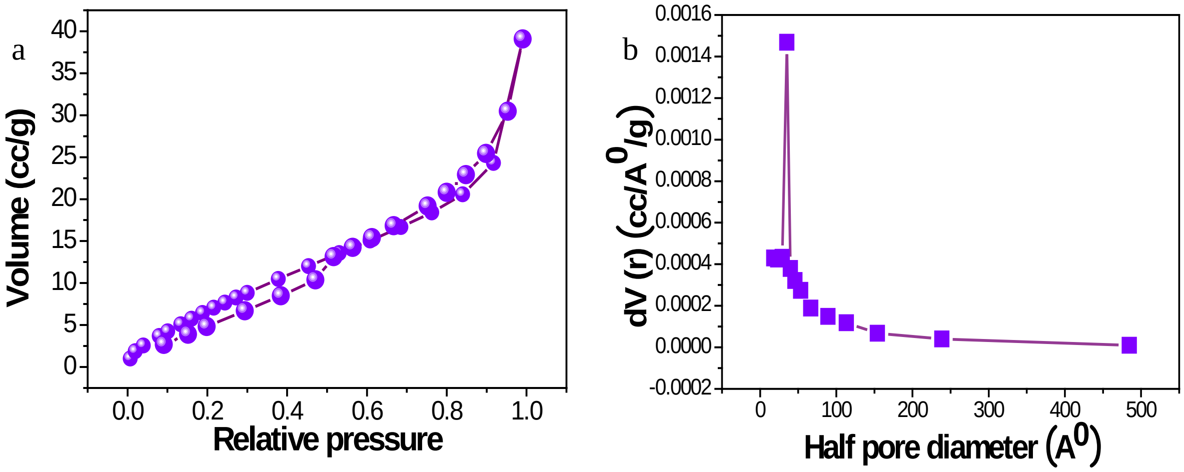

Figure S5. (a) BET Analysis and (b) pore size distribution of PRGO-nt.

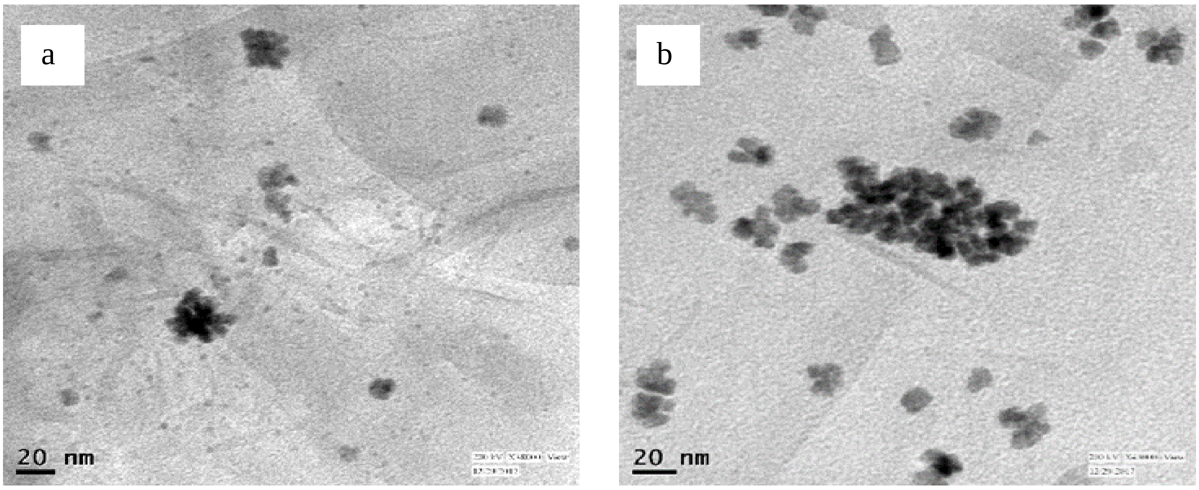

Figure S6. TEM images in different conditions (a) low BP (3×10^{-3} M) (b) high BP (3×10^{-2} M).

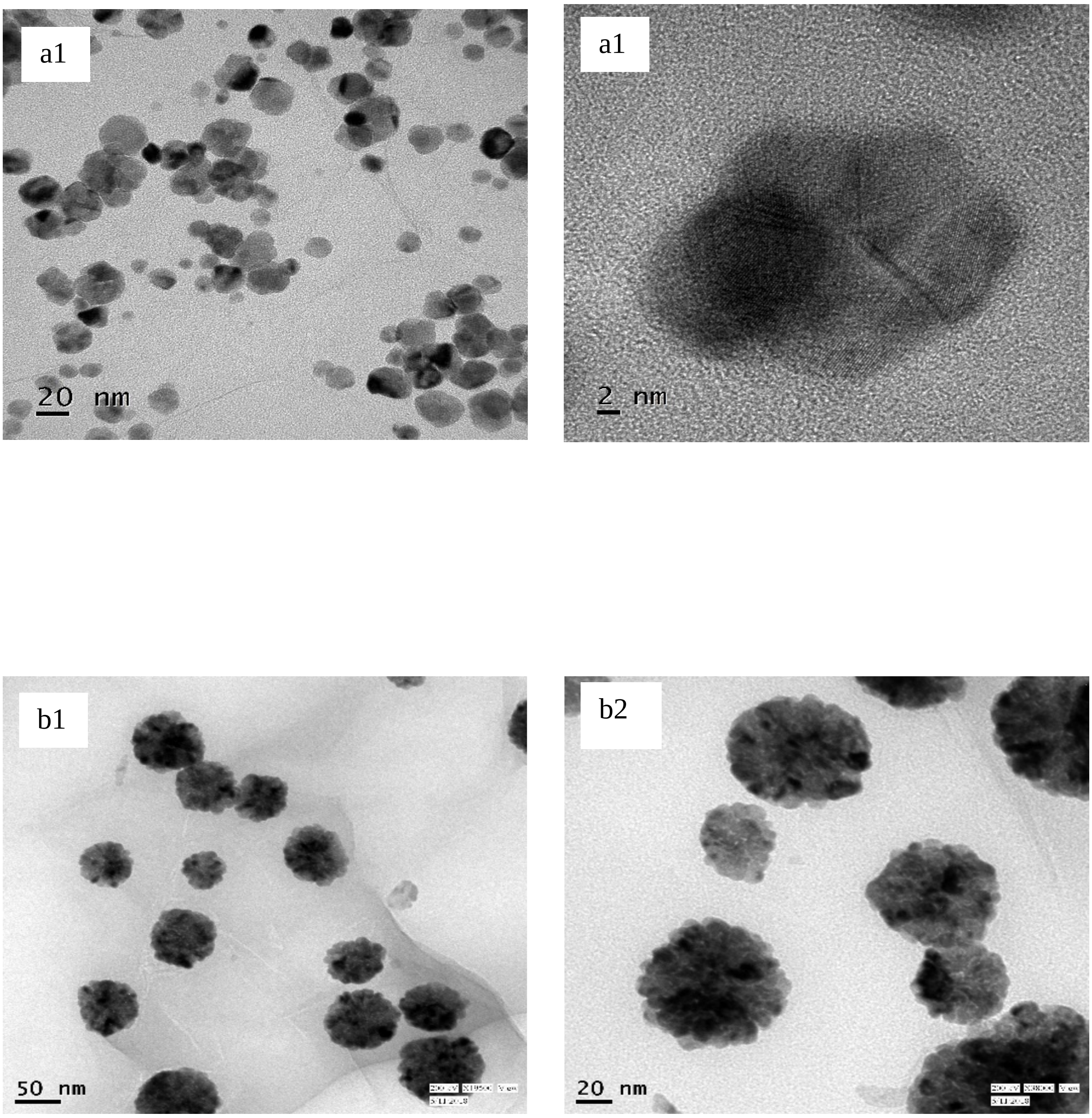
a1
20 nm
a1
2 nm
b1
50 nm
b2
20 nm

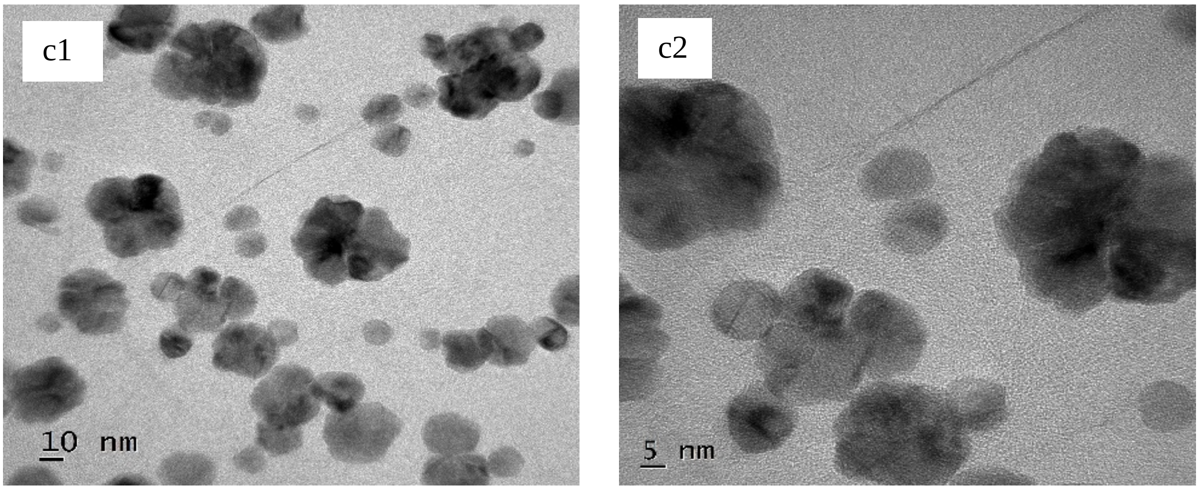

Figure S7. TEM images of Pd/rGO synthesized in substitution of BP, using (a) acetone (b) hydroquinone and (c) acetophenone.

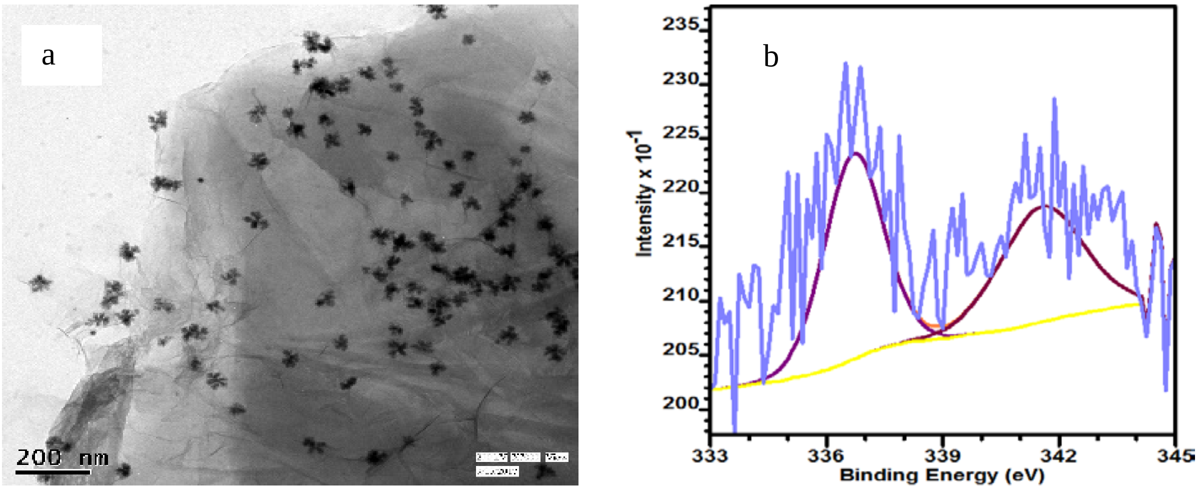

Figure S8. (a) TEM images, and (b) decreased intensity of Pd 3d XPS spectrum of used PRGO-nd.

Figure S9. NMR DATA

Bi-phenyl

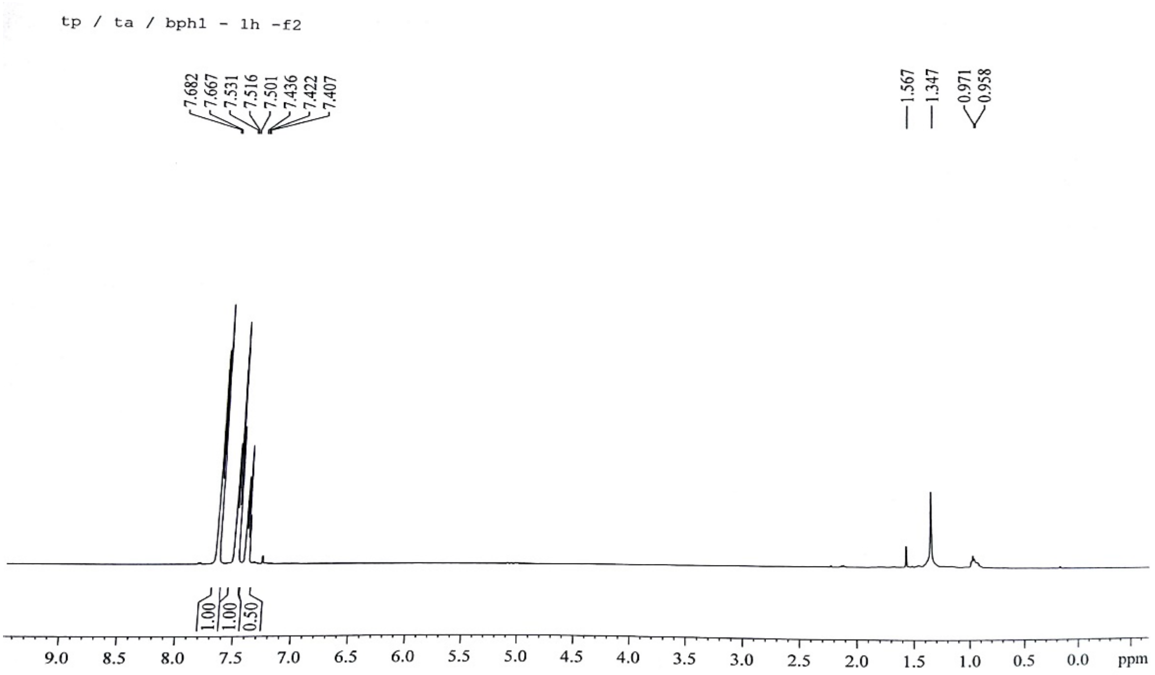

^{1}H (500 MHz, $CDCl_3$, ppm): δ 7.68 (d, J = 7.5 Hz, 4H), 7.53-7.50 (m, 4H), 7.44-7.41 (m, 2H)

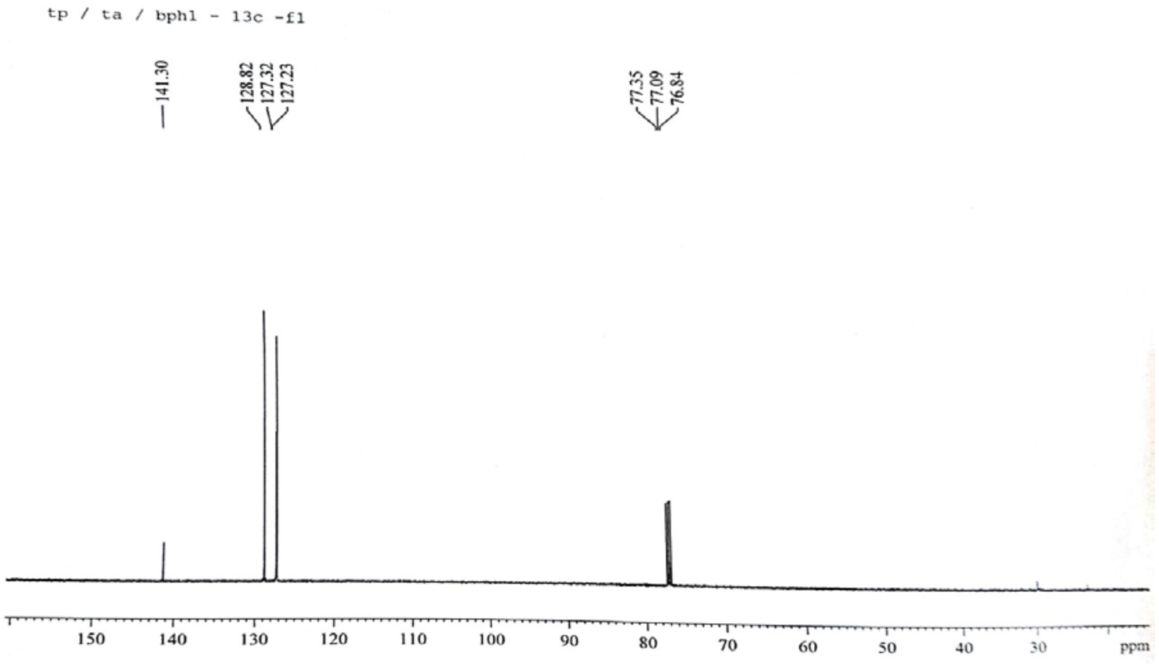

^{13}C (125 MHz, $CDCl_3$, ppm): δ = 141.3, 128.8, 127.3, 127.2

2-phenyltoluene

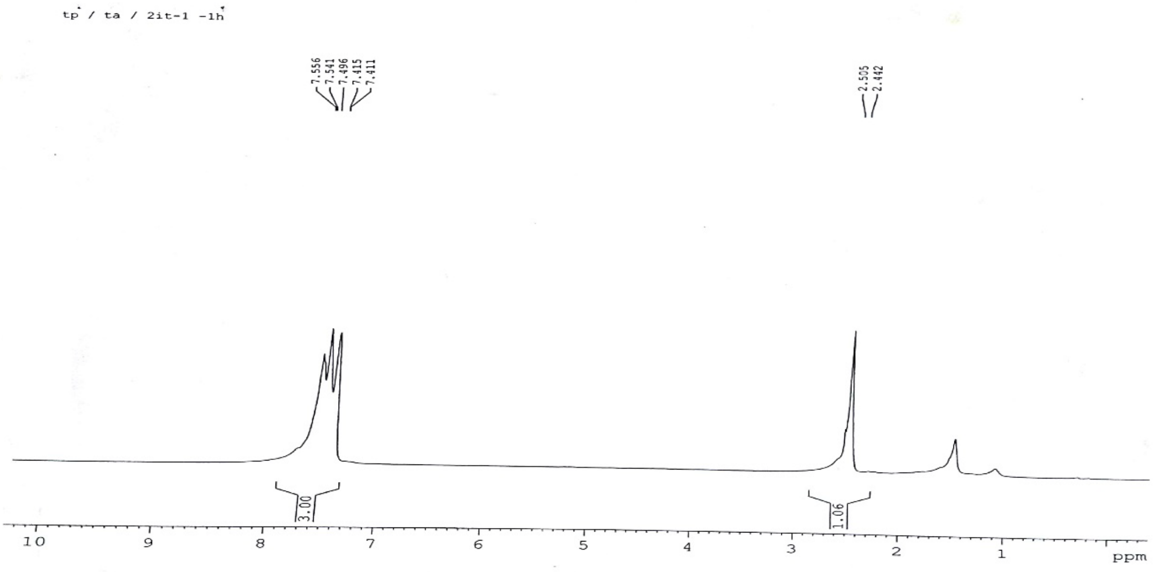

^{1}H (400 MHz, $CDCl_3$): δ 7.56-7.41 (m, 9H), 2.44 (s, 3H).

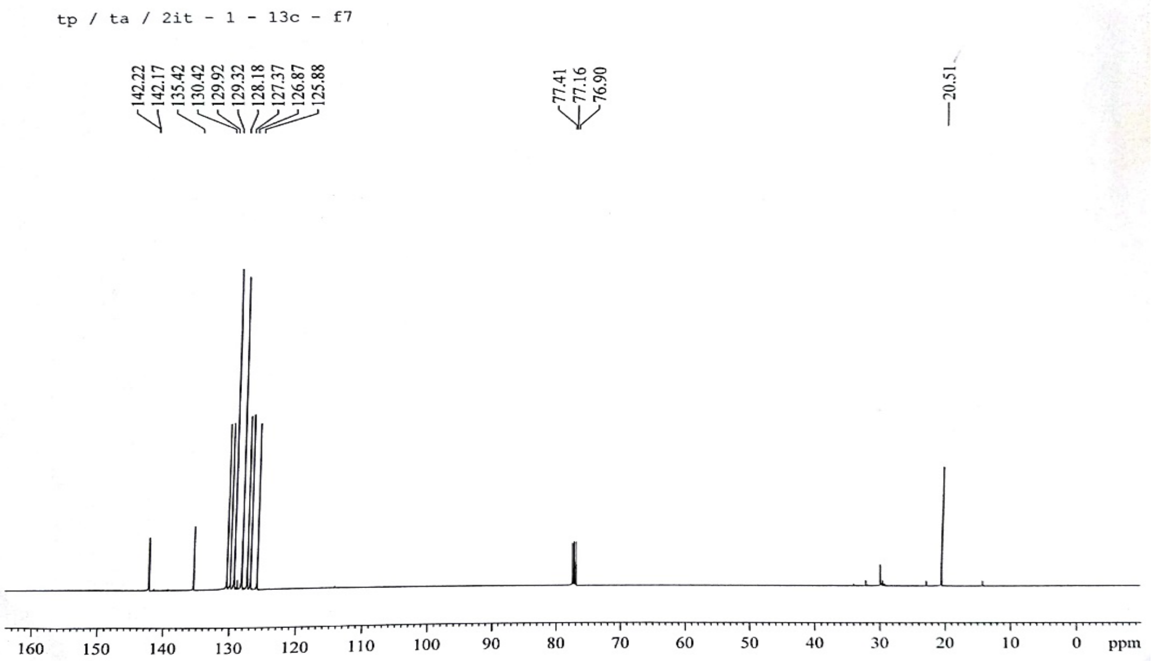

^{13}C (125 MHz, $CDCl_3$, ppm): δ = 142.2, 142.1, 135.4 129.9, 129.3, 128.2, 127.4, 126.9, 125.9, 20.51.

4-phenyltoluene

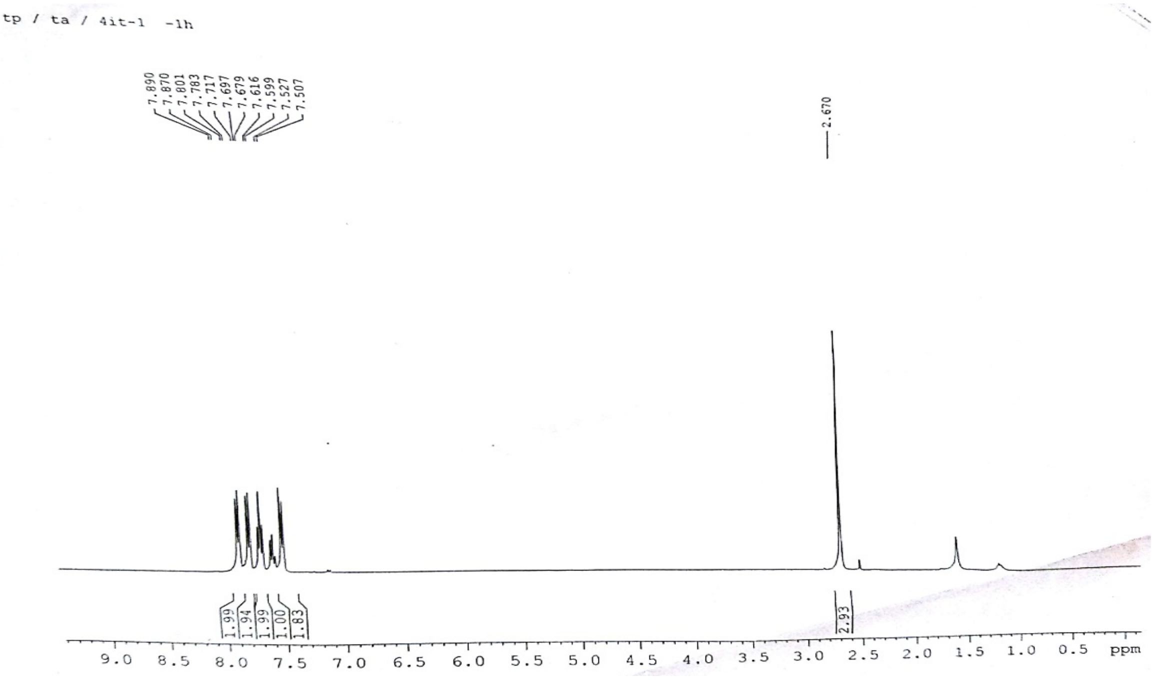

^{1}H (400 MHz, $CDCl_3$, ppm): δ 7.88 (d, 2H, J = 8 Hz), 7.79 (d, 2H, J = 7.2 Hz), 7.69 (t, 2H, J = 7.6 Hz), 7.61 (t, 1H, J = 7 Hz), 7.52 (d, 2H, J = 8 Hz), 2.67 (s, 3H)

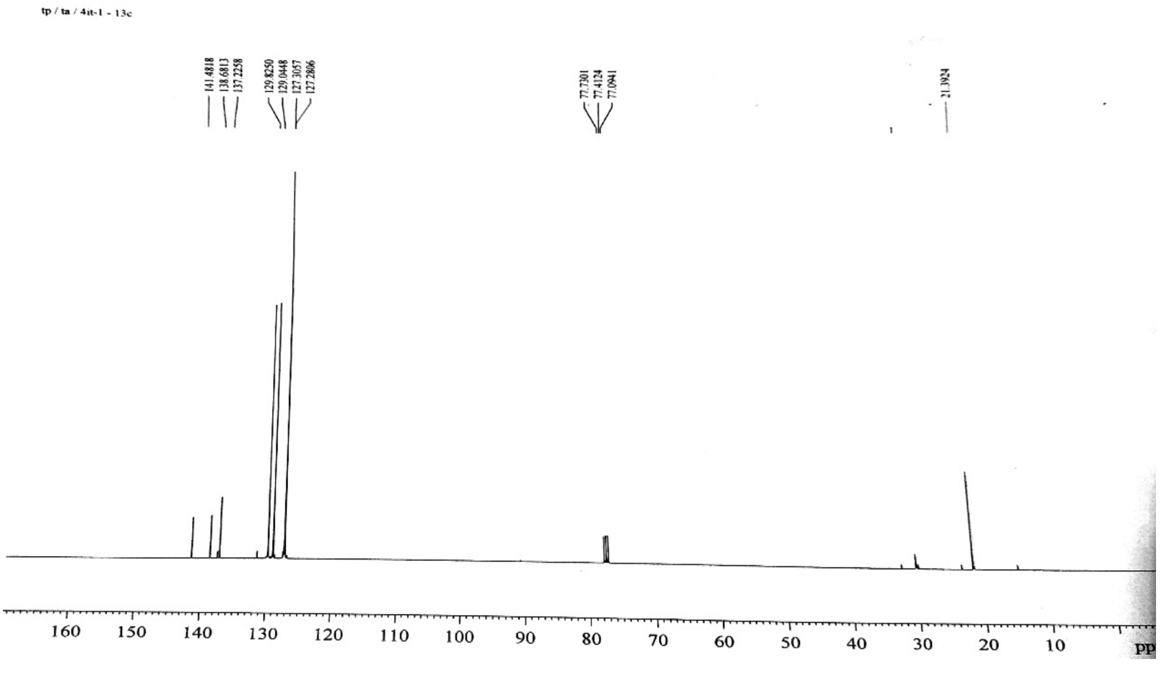

^{13}C (100 MHz, $CDCl_3$, ppm): δ = 141.5, 138.7, 137.2, 129.8, 129.0, 127.3, 127.3, 21.4.

4-phenylanisole

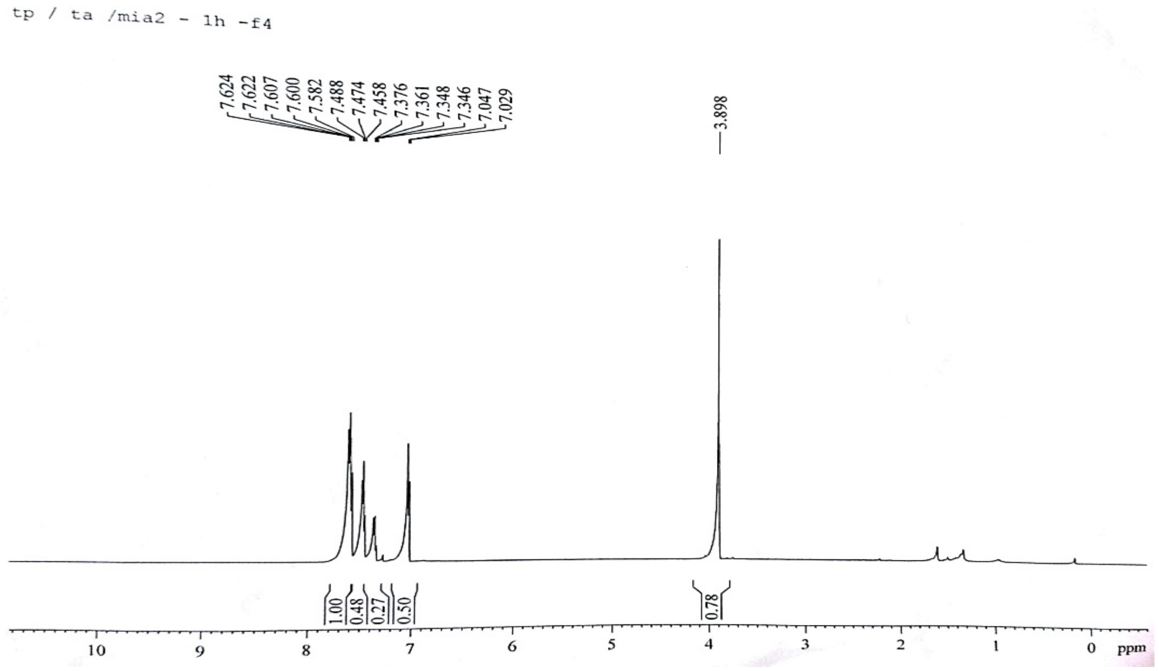

^{1}H (500 MHz, $CDCl_3$, ppm): δ 7.63-7.58 (m, 4H), 7.47 (t, 2H, J =7.5 Hz), 7.38-7.35 (m, 1H), 7.04 (d, 2H, J = 9 Hz), 3.89 (s, 3H)

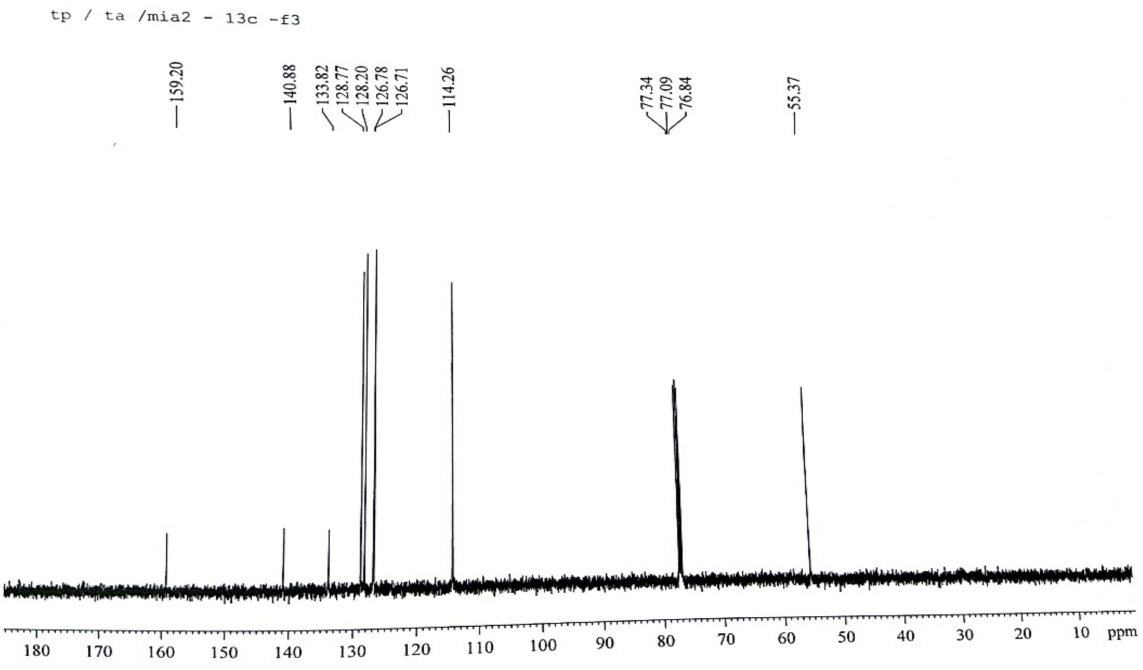

^{13}C (125 MHz, $CDCl_3$, ppm): δ = 159.2, 140.9, 133.8, 128.8, 128.2, 126.8, 126.7, 114.3, 55.4.

4-phenylchloronbenzene

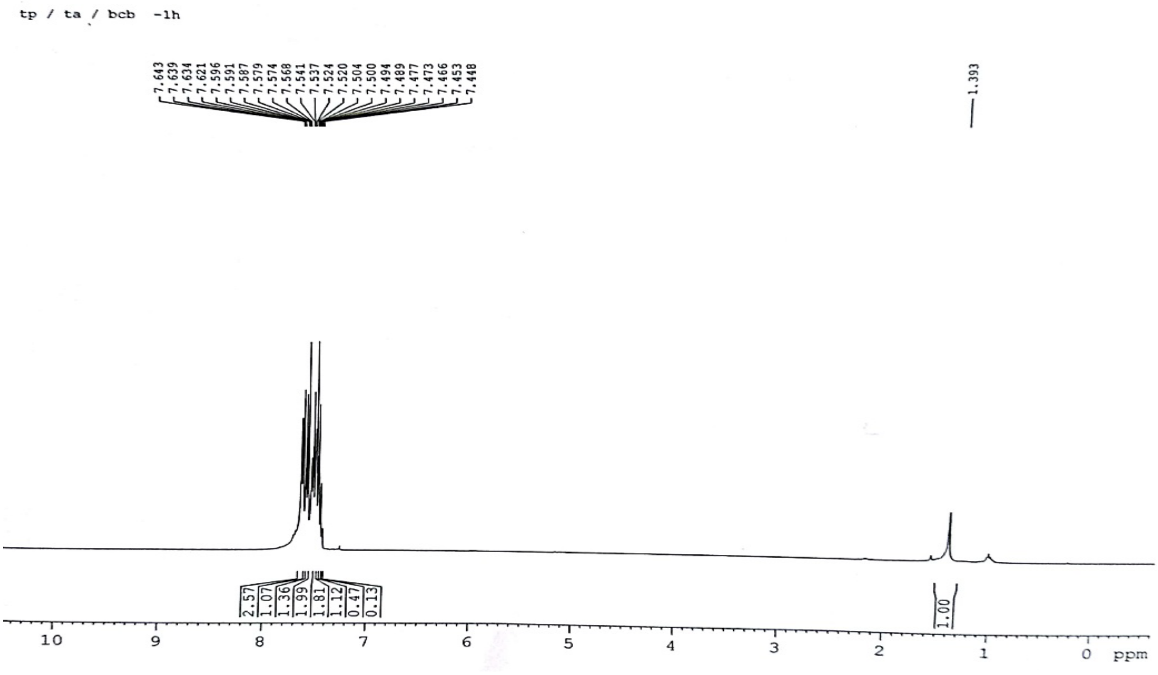

^{1}H (400 MHz, $CDCl_3$, ppm): δ 7.64-7.57 (m, 4H), 7.54-7.45 (m, 5H)

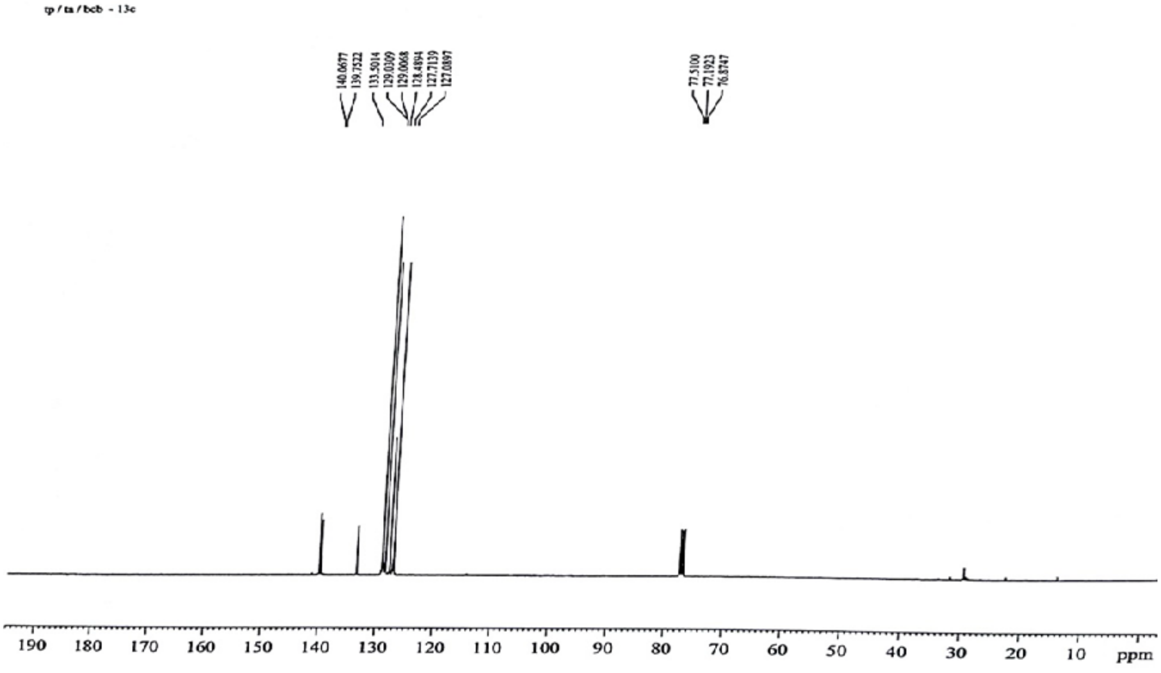

^{13}C (100 MHz, $CDCl_3$, ppm): δ = 140.1, 139.8, 133.5, 129.1, 129.0, 128.5, 127.7, 127.1

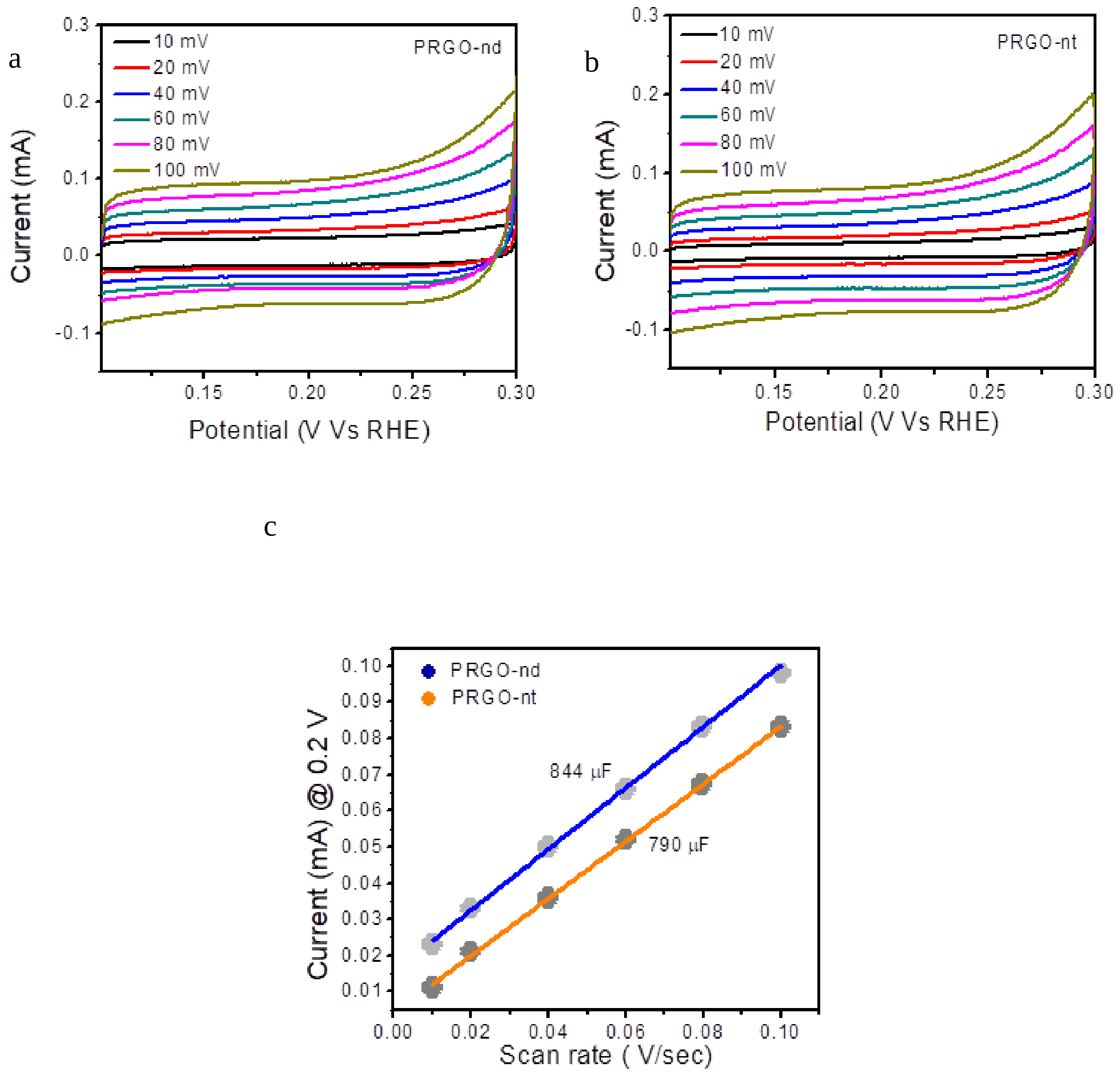

Figure S10. CVs at different scan rates in the potential range of 0.1-0.3 V (*vs.* RHE), within non-faradaic region, in 0.5 M H_2SO_4 solution with (a) PRGO-nd and (b) PRGO-nt. (c) Capacitive currents measured at 0.2 V (*vs.* RHE) as a function of scan rates with respective catalyst system.

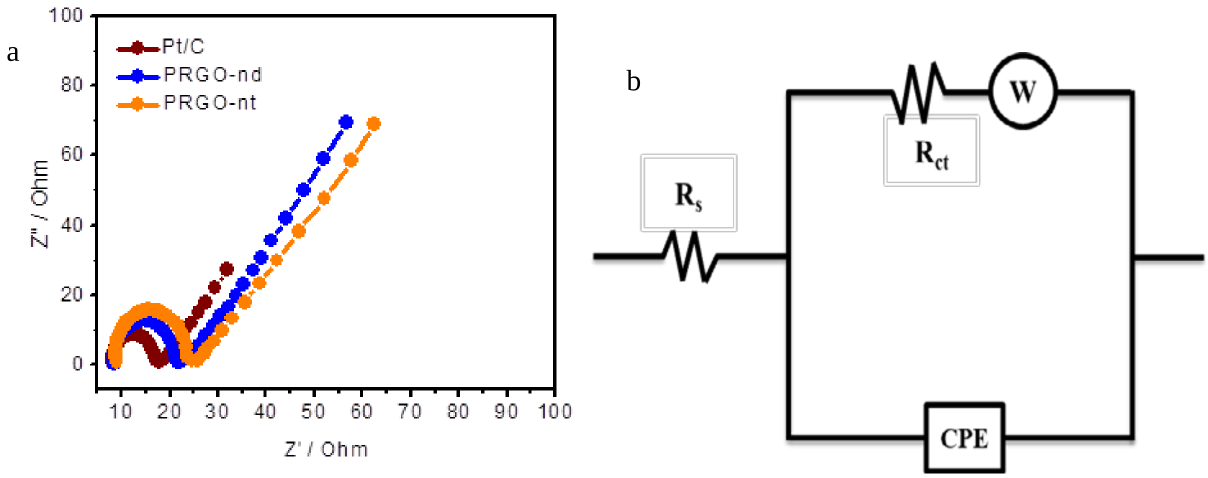

Figure S11. (a) Nyquist plot (from EIS) of as-synthesized catalysts, and commercial Pt/C examined at -0.068 V (*vs.* RHE). (b) Equivalent circuit diagram where CPE is the constant phase element, W is the Warburg element, R_s is the series resistance and R_{ct} denoted the charge transfer resistance.
